# High rumen degradable starch decreased goat milk fat via *trans*-10, * cis*-12 conjugated linoleic acid-mediated downregulation of lipogenesis genes, particularly, *INSIG1*

**DOI:** 10.1186/s40104-020-00436-3

**Published:** 2020-04-06

**Authors:** Lixin Zheng, Shengru Wu, Jing Shen, Xiaoying Han, Chunjia Jin, Xiaodong Chen, Shengguo Zhao, Yangchun Cao, Junhu Yao

**Affiliations:** 1 College of Animal Science and Technology, Northwest A&F University, Yangling, 712100 Shaanxi China; 2 State Key Laboratory of Animal Nutrition, Institute of Animal Sciences, Chinese Academy of Agricultural Sciences, Beijing, 100193 China

**Keywords:** Dairy goat, *INSIG1*, Milk fat depression, Rumen degradable starch, *Trans*-10, * cis*-12 CLA

## Abstract

**Background:**

Starch is an important substance that supplies energy to ruminants. To provide sufficient energy for high-yielding dairy ruminants, they are typically fed starch-enriched diets. However, starch-enriched diets have been proven to increase the risk of milk fat depression (MFD) in dairy cows. The starch present in ruminant diets could be divided into rumen-degradable starch (RDS) and rumen escaped starch (RES) according to their different degradation sites (rumen or intestine). Goats and cows have different sensitivities to MFD. Data regarding the potential roles of RDS in milk fat synthesis in the mammary tissue of dairy goats and in regulating the occurrence of MFD are limited.

**Results:**

Eighteen Guanzhong dairy goats (day in milk = 185 ± 12 d) with similar parity, weight, and milk yield were selected and randomly assigned to one of three groups (*n* = 6), which were fed an LRDS diet (Low RDS = 20.52%), MRDS diet (Medium RDS = 22.15%), or HRDS diet (High RDS = 24.88%) for 5 weeks. Compared with that of the LRDS group, the milk fat contents in the MRDS and HRDS groups significantly decreased. The yields of short-, medium- and long-chain fatty acids decreased in the HRDS group. Furthermore, increased RDS significantly decreased ruminal *B. fibrisolvens* and *Pseudobutyrivibrio* abundances and increased the *trans*-10, * cis*-12 conjugated linoleic acid (CLA) and *trans*-10 C18:1 contents in the rumen fluid.

A multiomics study revealed that the HRDS diet affected mammary lipid metabolism down-regulation of *ACSS2*, *MVD*, *AGPS*, *SCD5*, *FADS2*, *CERCAM*, *SC5D*, *HSD17B7*, *HSD17B12*, *ATM*, *TP53RK*, *GDF1* and *LOC102177400*. Remarkably, the significant decrease of *INSIG1*, whose expression was depressed by *trans*-10, * cis*-12 CLA, could reduce the activity of *SREBP* and, consequently, downregulate the downstream gene expression of SREBF1.

**Conclusions:**

HRDS-induced goat MFD resulted from the downregulation of genes involved in lipogenesis, particularly, *INSIG1*. Specifically, even though the total starch content and the concentrate-to-fiber ratio were the same as those of the high-RDS diet, the low and medium RDS diets did not cause MFD in lactating goats.

## Background

To provide sufficient energy for high-yielding dairy ruminants, they are typically fed starch-enriched diets. However, starch-enriched diets are known to increase the risk of milk fat depression (MFD) in dairy cows [1–3]. MFD is a multifactorial disorder characterized by sustained reduction in milk fat synthesis, which can reduce milk fat production and milk quality, induce potentially adverse effects on animal health and bring about important economic losses [4].

The starch present in ruminant diets could be divided into rumen-degradable starch (RDS) and rumen escaped starch (RES) according to their different degradation sites (rumen or intestine). The content of RDS depends on the total starch content of the diet and the ruminal starch degradability differences among grains [5]. Moreover, the amount of starch degradation in the rumen can be regulated, mainly by providing different sources of grains or altering processing methods of grains, which could maintain rumen health and prevent the occurrence of MFD [6]. Hence, it is possible to regulate the RDS content and maintain ruminal and mammary health when feeding dairy ruminants with a starch-enriched diet for high dairy yields.

A starch-enriched diet can lead to acid accumulation and low rumen pH, altering rumen biohydrogenation [7, 8]. Studies have shown that the occurrence of MFD is closely related to the intermediates of unsaturated fatty acid biohydrogenation in the rumen, including *trans*-10, * cis*-12 conjugated linoleic acid (CLA), * cis*-10, * trans*-12 CLA, *trans*-9, * cis*-11 CLA, *trans*-10 C18:1, etc. [9–11]. MFD-related intermediates, such as increased *trans*-10, * cis*-12 CLA, could inhibit mammary uptake and *de novo* synthesis of fatty acids, consequently decreasing milk fat production [12, 13].

In previous studies, the effects of nutritional regulation paradigms on milk fat synthesis and candidate genes related to lipid metabolism were evaluated [14, 15]. However, milk fat synthesis is a dynamic and complex multinetwork regulation process with a large number of involved genes [16] and needs further systematic study. Moreover, the effects of trans intermediates on milk fat synthesis in different species of mammary tissue are inconsistent [9, 17, 18]. Briefly, feeding lactation cows with a diet high in polyunsaturated fatty acids could downregulate the mRNA abundance of *ACACA, ACSS2* and *FASN* genes associated with *de novo* fatty acid synthesis, but this did not occur in lactating goats [19]. In addition, data regarding the potential roles of RDS in milk fat synthesis in the mammary tissue of dairy goats and in regulating the occurrence of MFD are limited.

The ruminal outflow of trans intermediates was shown to increase as the abundance of hydrogenated bacteria declined [20]. A previous study showed that changes in fatty acid hydrogenation products in the rumen were closely related to the rumen bacteria, *Butyrivibrio* [21]. The key *Butyrivibrio* spp. declined linearly with increasing unsaturated fatty acids. Hence, altered rumen bacteria involved in biohydrogenation could serve as main factors affecting milk fat synthesis. To elucidate the mechanism of RDS-induced MFD, it is necessary to analyze the response of rumen bacteria involved in the biohydrogenation of RDS.

One of the commonly used methods to increase dietary RDS is replacing corn with wheat because the degradation rate of wheat starch in the rumen is higher than that of corn starch [22]. Moreover, transcriptomic and metabolomic technologies provide opportunities to better understand the regulatory mechanisms of RDS on milk fat synthesis. Herein, by partially replacing corn with wheat and implementing different RDS diets, the present study aimed 1) to profile the transcriptional alterations of mammary tissue and metabolite changes of mammary venous blood in dairy goats fed different RDS diets, 2) to reveal the molecular mechanisms of RDS-induced MFD, and 3) to identify the major microbes involved in the biohydrogenation of rumen fatty acids in dairy goats.

## Materials and methods

### Animals, diets, experimental procedures and sample collection

Eighteen Guanzhong dairy goats (days in milk = 185 ± 12 d) were paired and blocked based on body weight and milk yield and then assigned to 1 of 3 groups (*n* = 6): the LRDS group (Low RDS = 20.52%), MRDS group (Medium RDS = 22.15%) and HRDS group (High RDS = 24.88%). To study the differential effects of different RDS levels on the occurrence of MFD and the underlying mechanism, three diets with different RDS levels were designed based on our previous study [22]. Dietary RDS was calculated using the following formula: RDS= $\sum \limits_{i=1}^n Pi$ ×ERD_*i*_, where P_*i*_ represents the proportion of dietary starch of feed *i* in the diet, ERD_*i*_ represents the effective starch degradability of feed *i*, and *n* is the number of ingredients containing starch in the feed formula [5]. The ERD parameters were calculated according to in situ ruminal degradation. A detailed method of in situ ruminal degradation was reported in Li et al. [23]. The forage-to-concentrate ratio of the 3 diets was 45:55, and the diets were formulated to be isoenergetic, isonitrogenous, and isostarch. The difference in dietary RDS was made by partially replacing corn with wheat. The details of the dietary components and chemical compositions are shown in Table S1. The goats were fed twice a day at 08:30  and 16:30  for *ad libitum* intake (allowing for 5–10% refusals). All goats were individually housed in tie-stall barns and had free access to water. This animal experiment lasted 5 weeks after 2 weeks of adaptation to the dietary regimen (LRDS diet). The feed intake was measured, and feed samples were collected before the morning feeding once a week. Each goat was milked individually twice a day at 08:00  and 16:00  using an electric milking machine, and two milk samples (2/3 from the morning and 1/3 from the evening milkings) were taken and pooled as a daily sample. Milk composition analysis was performed with a MilkoScan FT1 (FOSS, Denmark) and included the fat content, protein content, lactose content, milk urea nitrogen content, and somatic cell count (SCC). During the last week of the experiment, the milk yield of individual goats was recorded at each milking for 3 consecutive days.

At termination of the experiment, 3 h after morning feeding, mammary venous blood samples were collected. Then, all goats were euthanized by exsanguination after anesthesia using 0.1 mg/kg BW xylazine, 5 mg/kg BW ketamine, and 0.25 mg/kg BW diazepam as a single intravenous injection [24]. Blood samples were collected into 5 mL vacutainer tubes with the chelating agent EDTA-K_2_, and then the samples were centrifuged at 3,500 × *g* and 4 °C for 15 min to obtain plasma. Each aliquot (300 μL) of the plasma was stored at − 80 °C for further metabolomics analysis. Mammary tissues were aseptically sampled from the left rear quarter of the mammary gland and immediately preserved in liquid nitrogen until RNA extraction. Rumen fluid was collected and filtered through 4 layers of cheesecloth and then stored at − 80 °C for fatty acid analysis and DNA extraction.

### Chemical analysis

Feed samples were analyzed for dry matter (methods 934.01; AOAC, 1995), crude protein (CP, methods 976.05; AOAC, 1995), ether extract (methods 920.39; AOAC, 1995), neutral detergent fiber (NDF), acid detergent fiber (ADF) and starch (Megazyme, Bray, Ireland) [25]. Additionally, the feed and rumen fluid fatty acid profiles were analyzed using high-performance capillary gas chromatography (HPGC, 7820A, Agilent Technologies, Santa Clara, USA) with a flame ionization detector and a fused silica capillary column (CP-7420, 100 m × 0.25 mm × 0.25 μm) according to Sun and Gibbs [26]. The fatty acid composition of the milk was determined by HPGC with in accordance with Shi et al. [27]. Nonadecanoic acid was used as an internal standard, and a 37-component FAME mix, *cis*-9, * trans*-11 CLA, *trans*-10, * cis*-12 CLA (Sigma Chemical Co, Saint Louis, USA) and ME93 (Larodan Fine Chemicals AB, Malmo, Sweden) were used as external standards. All fatty acid composition results are expressed as g/100 g of total fatty acids.

### Metabolomic analysis

Approximately 100 μL of plasma was preprocessed for metabolomic analyses. The derivatives of the sample were analyzed using liquid chromatography-tandem mass spectrometry (LC-MS/MS), and the acquired data were processed as described in a previous study [28]. Metabolic profiling of mammary venous plasma was performed using an ultrahigh-performance liquid chromatography (UHPLC) instrument (1290 Infinity LC, Agilent Technologies) coupled to triple time-of-flight mass spectrometer (AB Sciex TripleTOF 5600) at Shanghai Applied Protein Technology Co., Ltd. Metabolic pathway analysis of the identified metabolites in MRDS vs. LRDS, HRDS vs. LRDS and HRDS vs. MRDS were determined using the Kyoto Encyclopedia of Genes and Genomes (KEGG) pathway database (http://www.genome.jp/kegg/). Statistical analysis for pathway enrichment was performed using Fisher’s exact test, and significantly different pathways were defined with *P* < 0.05 [29].

### Transcriptomics analysis

Total mammary tissue RNA was extracted using RNAiso Plus reagent (Takara, Dalian, China), and genomic DNA was removed using DNase I. Only high-quality RNA samples (OD_260/280_ > 1.8, OD_260/230_ > 2.0, and the RNA integrity number > 8) were further sequenced (Shanghai Majorbio Biotechnology Co. Ltd., China). Approximately 5 μg of total RNA was used to prepare an mRNA library for paired-end sequencing (2 × 150 bp read length) on a NovaSeq sequencing system (Illumina HiSeq. 4000, San Diego, USA) according to a previous study [30]. The capra-hircus gene annotation list was used as background (https://www.ncbi.nlm.nih.gov/genome/?term=txid9925[orgn]). To identify differentially expressed genes (DEGs) between samples from the compared groups, including MRDS vs. LRDS, HRDS vs. LRDS, and HRDS vs. MRDS, the expression level of each transcript was calculated as the fragments per kilobase of exon per million mapped reads (FRKM) by using RSEM software (http://deweylab.biostat.wisc.edu/rsem/). The R statistical package DESeq2 was used to screen out the DEGs with a false discovery rate (FDR) value < 0.1 and a fold change (FC) greater than 1.5 [31, 32]. Gene Ontology (GO) functional enrichment and KEGG pathway analysis were carried out by Goatools (https://github.com/tanghaibao/Goatools) and KOBAS (http://kobas.cbi.pku.edu.cn/home.do), and the significance threshold was set as *P* < 0.05.

### Construction of the WGCNA coexpression network

Weighted gene coexpression network analysis (WGCNA) of DEGs in goat mammary tissue was performed using R Software (v.3.3) and the WGCNA Package (v.1.67). The WGCNA method [33] was used to construct the coexpression network of the DEGs between samples from the compared groups, including MRDS vs. LRDS, HRDS vs. LRDS, and HRDS vs. MRDS. WGCNA clustered genes that had similar expression profiles into the same module with the software default parameters (soft threshold = 6, minModuleSize = 30). The milk fat content was considered the phenotype, and the correlations between eigengene modules and milk fat content were analyzed by Spearman correlation tests. The first 20 nodes in the module with the highest phenotypic correlation were selected for analysis, and the connections with weights greater than 0.02 between nodes were analyzed. Finally, the results were imported into the Cytoscape software (v.3.6.1, https://cytoscape.org/) for visual analysis.

### Real-time quantitative PCR

To validate the RNA-Seq gene expression pattern, the expression levels of six genes (*ACSS2*, *INSIG1*, *MVD*, *PNPLA3*, *SLC7A1* and *DHCR24*) associated with lipid metabolism were analyzed using qRT-PCR. Total RNA (1,000 ng) was reverse transcribed using a Prime Script® RT reagent kit (Takara, China) according to the standard procedures of the manufacturer. Quantification was performed with iCycler IQTM5 (Bio-Rad, USA) using SYBR® Premix Ex TaqTM II (Takara, China). A total of a 20-μL reaction system comprised the following solutions: 1 μL cDNA, 1 μL forward primer (10 pmol/μL), 1 μL reverse primer (10 pmol/μL), 10 μL SYBR Premix Ex Taq, and 7 μL nuclease-free water. The PCR protocol was as follows: 95 °C for 30 s, followed by 40 cycles of 95 °C for 5 s, 60 °C for 30 s, and 72 °C for 30 s, with a final extension at 72 °C for 30 s. Melting curve analysis and gel electrophoresis were performed after PCR amplification to ensure primer specificity and a single PCR product. The relative stability of the three housekeeping genes (*GAPDH*, *UXT* and *MRPL39*) was calculated by GeNorm software [34], and the most stable gene (*GAPDH*) was used as an internal normalization control. The specific primers for the qRT-PCR of *GAPDH* and tested mRNAs are listed in Table S2. All samples were examined in triplicate and analyzed using the 2^−ΔΔCt^ method [35].

### DNA extraction and qPCR of the rumen bacterial population

Metagenomic DNA from 3 mL of rumen fluid was extracted using the modified cetyltrimethyl ammonium bromide (CTAB) method [36]. The integrity of the DNA was assessed using 1% agarose gel electrophoresis, and the purity was assessed with a 260:280 nm ratio (> 1.8) using a NanoDrop ND2000 spectrophotometer (NanoDrop Technologies Inc., DE, USA). By using the population of total eubacteria as an internal reference, the relative population abundances of specific eubacteria were detected by using qPCR and expressed as the percentage of the total eubacterial 16S rDNA gene in accordance with the protocol of Shingfield et al. [20]. Additionally, the specificity of the primers (Table S3) for the amplification of the 16S sequences of differential eubacteria was proven by Shingfield et al. [20]. The 2^−ΔCt^ method was used for calculating the relative expression of the tested bacteria [37].

### Statistical analysis

Except for the omics data, statistical evaluation was performed by variance analysis using a randomized experimental design (paired and blocked based on body weight and milk yield). If a significant treatment effect was observed by the variance analysis, the significance of the differences between treatments was determined using Duncan’s multiple comparisons test. All data are expressed as the mean and standard error. Significance was defined as *P* < 0.05, and trends were defined as 0.05 ≤ *P* < 0.10.

## Results

### High RDS diet can significantly reduce milk fat content and yield

The effects of the different feeds with 3 dietary RDS levels on dry matter intake and milk production and composition were measured (Table 1). No effect of RDS on dry matter intake was identified in the present study. The lactation efficiency (FPCM/DMI) tended to increase in the MRDS group (*P* < 0.1), while the milk yield tended to decrease in the HRDS group (*P* < 0.1). Compared with the that in the milk from the LRDS and MRDS groups, the SCC significantly increased in the milk from the HRDS group. Moreover, the milk fat content and yield significantly decreased in the HRDS group compared with those in the LRDS group (*P* < 0.01). Although the milk fat content was significantly lower in the MRDS group than in the LRDS group, the milk fat yield was not significantly different from that of the LRDS group. Furthermore, the concentrations of major milk fatty acids in the three treatment groups were determined and are shown in Table 2. Compared with those of the LRDS group, dietary MRDS and HRDS supplementation exhibited significantly reduced concentrations of *trans*-9 C18:1 and *cis*-9 C18:1 in milk. Specifically, the proportion of *trans*-10, * cis*-12 CLA significantly increased in the HRDS group compared with that in the LRDS group. Furthermore, the HRDS group showed a significantly sharp decline in the yield of short (*P* = 0.013), medium (*P* = 0.025) and long chain fatty acids (*P* = 0.012) in milk compared with those in the milk of the LRDS and MRDS groups (Table 3). Overall, the above results indicated that the HRDS diet induced MFD, while the LRDS and MRDS groups did not induce MFD.

**Table 1:** Lactation performance of dairy goats fed diets with different RDS contents

Item	Treatment^c^			SEM^d^	*P*-value
	LRDS	MRDS	HRDS		
DMI, kg/d	2.0	2.1	2.1	0.02	0.68
Milk yield, kg/d	1.4	1.7	1.2	0.11	0.09
FPCM^e^, kg/d	1.4	1.5	1.0	0.09	0.06
Milk composition, %					
Fat	3.74^a^	3.06^b^	3.01^b^	0.11	<0.01
Protein	2.95	3.04	2.98	0.06	0.85
Lactose	4.70	4.57	4.57	0.06	0.58
MUN, mg/dL	31.47	30.05	30.40	1.21	0.89
SCC, 10^3^/mL	392^b^	640^b^	2271^a^	339	0.04
Milk composition yield, g/d					
Fat	53.6^a^	52.7^a^	32.1^b^	3.5	<0.01
Protein	44.8^ab^	51.5^a^	35.2^b^	2.7	0.03
Lactose	73.0	79.0	53.9	4.6	0.06
Efficiency, kg/kg					
Milk/DMI	0.71	0.82	0.57	0.05	0.11
FPCM/DMI	0.67	0.71	0.50	0.04	0.07

^a-b^ Means within the same row with different superscripts differ significantly (*P* < 0.05)

^c^Treatments were the LRDS diet (RDS = 20.52%), MRDS diet (RDS = 22.15%), and HRDS diet (RDS = 24.88%) with similar total starch contents

^d^*SEM* Standard error of mean

^e^*FPCM* fat-and protein-corrected milk yield

**Table 2:** Concentration of major fatty acids in the milk of goats fed different RDS diets

FAs, g/100 g of total FAs	Treatment^d^			SEM^e^	*P*-value
	LRDS	MRDS	HRDS		
C4:0	0.98	1.15	1.10	0.10	0.806
C6:0	1.55	1.68	1.33	0.12	0.524
C8:0	2.04	2.40	1.94	0.16	0.487
C10:0	8.11	9.59	8.92	0.51	0.519
C11:0	0.13	0.15	0.13	0.14	0.694
C12:0	4.91	6.51	6.20	0.35	0.142
C13:0	0.13	0.14	0.15	0.01	0.595
C14:0	11.15	13.13	11.09	0.49	0.156
*cis*-9 C14:1	0.20	0.22	0.23	0.02	0.463
C15:0	0.94	0.97	0.93	0.03	0.829
*cis*-10 C15:1	0.27	0.26	0.33	0.02	0.145
C16:0	30.14	30.79	31.63	0.81	0.773
*cis*-9 C16:1	1.23^b^	1.15^b^	1.48^a^	0.05	0.004
C17:0	1.20	1.20	1.39	0.05	0.273
*cis*-10 C17:1	0.42^b^	0.38^c^	0.48^a^	0.01	<0.001
C18:0	7.48	7.35	6.04	0.37	0.217
*cis*-9 C18:1	21.11^a^	16.25^b^	18.33^b^	0.62	0.001
*trans*-9 C18:1	0.27^a^	0.21^b^	0.19^b^	0.01	0.011
*trans*-10 C18:1	0.17	0.20	0.24	0.02	0.498
*trans*-11 C18:1	1.04	0.96	0.93	0.04	0.405
*cis*-9,12 C18:2	2.60	3.00	3.26	0.12	0.078
*trans*-9,12 C18:2	0.06	0.05	0.06	0.01	0.298
*cis*-9, * trans*-11 CLA	1.51	1.42	1.13	0.11	0.362
*trans*-10, * cis*-12 CLA	0.02^b^	0.06^ab^	0.13^a^	0.01	0.007
C18:3n-3	0.18	0.13	0.21	0.03	0.414
C18:3n-6	0.10	0.07	0.10	0.01	0.249
SFAs^f^	71.78^b^	76.65^a^	71.89^b^	0.82	0.012
MUFAs^g^	23.80^a^	18.71^c^	21.36^b^	0.65	0.001
PUFAs^h^	4.94	5.07	5.34	0.19	0.713

^a-c^ Means within the same row with different superscripts differ significantly (*P* < 0.05)

^d^Treatments were the LRDS diet (RDS = 20.52%), MRDS diet (RDS = 22.15%), and HRDS diet (RDS = 24.88%) with similar total starch contents

^e^*SEM* Standard error of mean

^f^*SFAs* Saturated fatty acids

^g^*MUFAs* Monounsaturated fatty acids

^h^*PUFAs* Polyunsaturated fatty acids

**Table 3:** Yield of major fatty acids in milk of dairy goats fed different RDS diets

FA yield^d^, g/d	Treatment^c^			SEM^e^	*P*-value
	LRDS	MRDS	HRDS		
C4:0	0.42	0.43	0.33	0.026	0.187
C6:0	0.70^a^	0.66^a^	0.42^b^	0.044	0.008
C8:0	1.03^a^	0.99^a^	0.62^b^	0.072	0.024
C10:0	4.08^a^	4.09^a^	2.77^b^	0.261	0.049
C11:0	0.64^ab^	0.73^a^	0.04^b^	0.006	0.052
C12:0	2.14^b^	3.08^a^	1.90^b^	0.176	0.007
C13:0	0.07^a^	0.07^a^	0.04^b^	0.005	0.021
C14:0	5.67^a^	6.46^a^	3.41^b^	0.465	0.011
*cis*-9 C14:1	0.10	0.11	0.07	0.009	0.229
C15:0	0.48^a^	0.49^a^	0.29^b^	0.004	0.033
*cis*-10 C15:1	0.13	0.11	0.10	0.008	0.277
C16:0	15.31^a^	15.59^a^	9.47^b^	1.114	0.029
*cis*-9 C16:1	0.63	0.58	0.42	0.040	0.091
C17:0	0.61	0.61	0.45	0.042	0.233
*cis*-10 C17:1	0.21	0.19	0.15	0.013	0.091
C18:0	3.77^a^	3.72^a^	1.85^b^	0.318	0.009
*cis*-9 C18:1	10.70^a^	8.17^ab^	5.55^b^	0.706	0.004
*trans*-9 C18:1	0.14^a^	0.10^a^	0.06^b^	0.010	0.002
*trans*-10 C18:1	0.09	0.11	0.07	0.013	0.577
*trans*-11 C18:1	0.55^a^	0.51^a^	0.30^b^	0.040	0.009
*cis*-9,12 C18:2	1.41	1.54	1.06	0.103	0.150
*trans*-9,12 C18:2	0.03	0.02	0.02	0.002	0.125
*cis*-9, * trans*-11 CLA	0.73^a^	0.70^a^	0.34^b^	0.071	0.035
*trans*-10, * cis*-12 CLA	0.01^b^	0.03^a^	0.03^a^	0.003	0.018
C18:3n-3	0.08	0.06	0.06	0.008	0.677
C18:3n-6	0.05	0.03	0.03	0.004	0.207
SCFAs^f^	1.13^a^	1.10^a^	0.75^b^	0.063	0.013
MCFAs^g^	7.32^ab^	8.27^a^	5.33^b^	0.472	0.025
LCFAsh	40.28^a^	38.60^a^	23.53^b^	2.734	0.012
SFAs^i^	38.73^a^	38.03^a^	21.88^b^	2.515	0.002
MUFAs^j^	12.06^a^	9.40^ab^	6.47^b^	0.784	0.006
PUFAs^k^	2.47	2.57	1.73	0.175	0.095

^a-b^ Means within the same row with different superscripts differ significantly (*P <* 0.05)

^c^Treatments were the LRDS diet (RDS = 20.52%), MRDS diet (RDS = 22.15%), and HRDS diet (RDS = 24.88%) with similar total starch contents

^d^Yield of major fatty acids = milk fat mass × individual fatty acid proportion

^e^*SEM* Standard error of mean

^f^*SCFAs* short chain fatty acids

^g^*MCFAs* medium chain fatty acids

^h^*LCFAs* long chain fatty acids

^i^*SFAs* saturated fatty acids

^j^*MUFAs* monounsaturated fatty acids

^k^*PUFAs* polyunsaturated fatty acids

The fatty acid composition of different feed samples from the 3 groups was further identified (Table S4), and the results showed that *cis*-9 C18:1 and C18:2n-6 were the predominant fatty acids. Moreover, along with increasing RDS level, the content of *cis*-9 C18:1 in the feed significantly decreased in the HRDS group compared with that in the LRDS group (*P* < 0.01), which was in accordance with the change in *cis*-9 C18:1 content in the milk when comparing groups HRDS and LRDS. Except for *cis*-9 C18:1 in feeds, other monounsaturated fatty acids in the LRDS and MRDS groups were significantly higher than those in the HRDS group. However, the content of *cis*-9,12 C18:2, which is the main resource of altered *trans*-10, * cis*-12 CLA in milk, was unchanged in the feed samples of the different groups.

### High RDS diet can significantly reduce the abundance of *B. fibrisolvens + Pseudobutyrvibrio*, which are involved in fatty acid biohydrogenation

The content of *cis*-9 C18:1 in the rumen fluid decreased with increasing RDS level (Table 4), which was also consistent with the trend of the *cis*-9 C18:1 content in the feed. The proportion of *trans*-10, * cis*-12 CLA was significantly higher in the HRDS group than in the LRDS and MRDS groups (*P* < 0.05). The proportion of *trans*-10 C18:1 was also significantly higher in the HRDS group than in the LRDS and MRDS groups (*P* < 0.05). The proportion of *trans*-11 C18:1 was not significantly different among the three groups. Moreover, the content of *cis*-9,12 C18:2, which served as the precursor of *trans*-10, * cis*-12 CLA, was also higher in the HRDS group than in the LRDS group (Table 4).

**Table 4:** Concentration of major fatty acids in rumen fluid of dairy goats fed different RDS diets

FA, g/100 g of total FA	Treatment^d^			SEM^e^	*P*-value
	LRDS	MRDS	HRDS		
C4:0	1.35	1.69	1.22	0.10	0.110
C6:0	0.24	0.28	0.16	0.25	0.146
C8:0	0.03	0.03	0.02	0.01	0.186
C10:0	0.12	0.12	0.11	0.01	0.909
C11:0	0.29^a^	0.25^ab^	0.21^b^	0.01	0.039
C12:0	0.83	1.04	0.86	0.04	0.113
C13:0	0.45	0.40	0.59	0.07	0.530
C14:0	3.37	3.18	3.38	0.20	0.906
*cis*-9 C14:1	3.17	3.94	3.77	0.20	0.291
C15:0	2.46	2.27	2.13	0.08	0.288
*cis*-10 C15:1	0.20	0.20	0.25	0.01	0.288
C16:0	32.43	32.45	32.83	0.23	0.761
*cis*-9 C16:1	1.14	1.31	1.33	0.04	0.113
C17:0	1.29^b^	1.54^a^	1.56^a^	0.05	0.018
*cis*-10 C17:1	0.21^b^	0.21^b^	0.26^a^	0.01	0.039
C18:0	16.64	17.01	15.69	0.64	0.715
*trans*-9 C18:1	0.28	0.31	0.31	0.01	0.695
*cis*-9 C18:1	12.64^a^	11.57^ab^	11.00^b^	0.27	0.033
*trans*-10 C18:1	0.19^b^	0.21^b^	0.43^a^	0.04	0.040
*trans*-11 C18:1	1.25	1.17	1.13	0.04	0.412
*cis*-9,12 C18:2	16.2^b^	18.1^a^	19.4^a^	0.02	0.025
*trans*-9,12 C18:2	0.28	0.16	0.22	0.09	0.041
*cis*-9, * trans*-11 CLA	0.17^b^	1.42^a^	1.13^ab^	0.02	0.076
*trans*-10, * cis*-12 CLA	0.11^b^	0.23^b^	0.65^a^	0.09	0.041
C18:3n-3	0.12	0.10	0.07	0.01	0.163
C18:3n-6	0.08	0.08	0.09	0.01	0.689
SFAs^f^	63.74	64.49	63.82	0.82	0.768
MUFAs^g^	17.99^a^	18.17^a^	16.66^b^	0.65	0.015
PUFAs^h^	17.27^c^	19.22^b^	20.64^a^	0.19	0.001

^a-c^ Means within the same row with different superscripts differ significantly (*P* < 0.05)

^d^Treatments were the LRDS diet (RDS = 20.52%), MRDS diet (RDS = 22.15%), and HRDS diet (RDS = 24.88%) with similar total starch contents

^e^SEM = Standard Error of Mean

^f^SFAs = saturated fatty acids

^g^MUFAs = monounsaturated fatty acids

^h^PUFAs = polyunsaturated fatty acids

The abundances of the major eubacteria known to be involved in fatty acid biohydrogenation in the rumen were examined using qPCR (Table 5). The different levels of RDS did not influence the abundance of *B. proteoclasticus, B. hungatei,* or *S. bovis* in the rumen fluid but significantly decreased the abundance of *B. fibrisolvens* + *Pseudobutyrivibrio* in the MRDS and HRDS groups. Moreover, the abundance of *P. acnes* tended to be greater in the HRDS group than in the other two groups (*P* < 0.1).

**Table 5:** Rumen microbial populations in dairy goats fed different RDS diets

Population, % of total eubacteria^d^	Treatment^c^			SEM^e^	*P*-value
	LRDS	MRDS	HRDS		
*B.fibrisolvens + Pseudobutyrivibrio*	0.0146^a^	0.0014^b^	0.0003^b^	0.00239	0.01
*B. proteoclasticus*	0.021	0.028	0.031	0.004	0.78
*B. hungatei*	0.0005	0.0003	0.0002	0.00008	0.48
*S. bovis*	0.004	0.002	0.002	0.0007	0.13
*P. acnes, n* × *10*^*−6*^	2.28	2.85	4.04	0.334	0.06

^a-b^ Means within the same row with different superscripts differ significantly (*P* < 0.05)

^c^Treatments were the LRDS diet (RDS = 20.52%), MRDS diet (RDS = 22.15%), and HRDS diet (RDS = 24.88%) with similar total starch contents

^d^Populations determined based on quantitative PCR using primers designed to target different members of *Butyrivibrio/Pseudobutyrivibrio* group, *S. bovis*, and *P. acnes*

^e^*SEM* Standard error of mean

### High RDS diet disturbed fatty acid and primary bile acid synthesis in goat mammary tissue

**Fig. 1:**
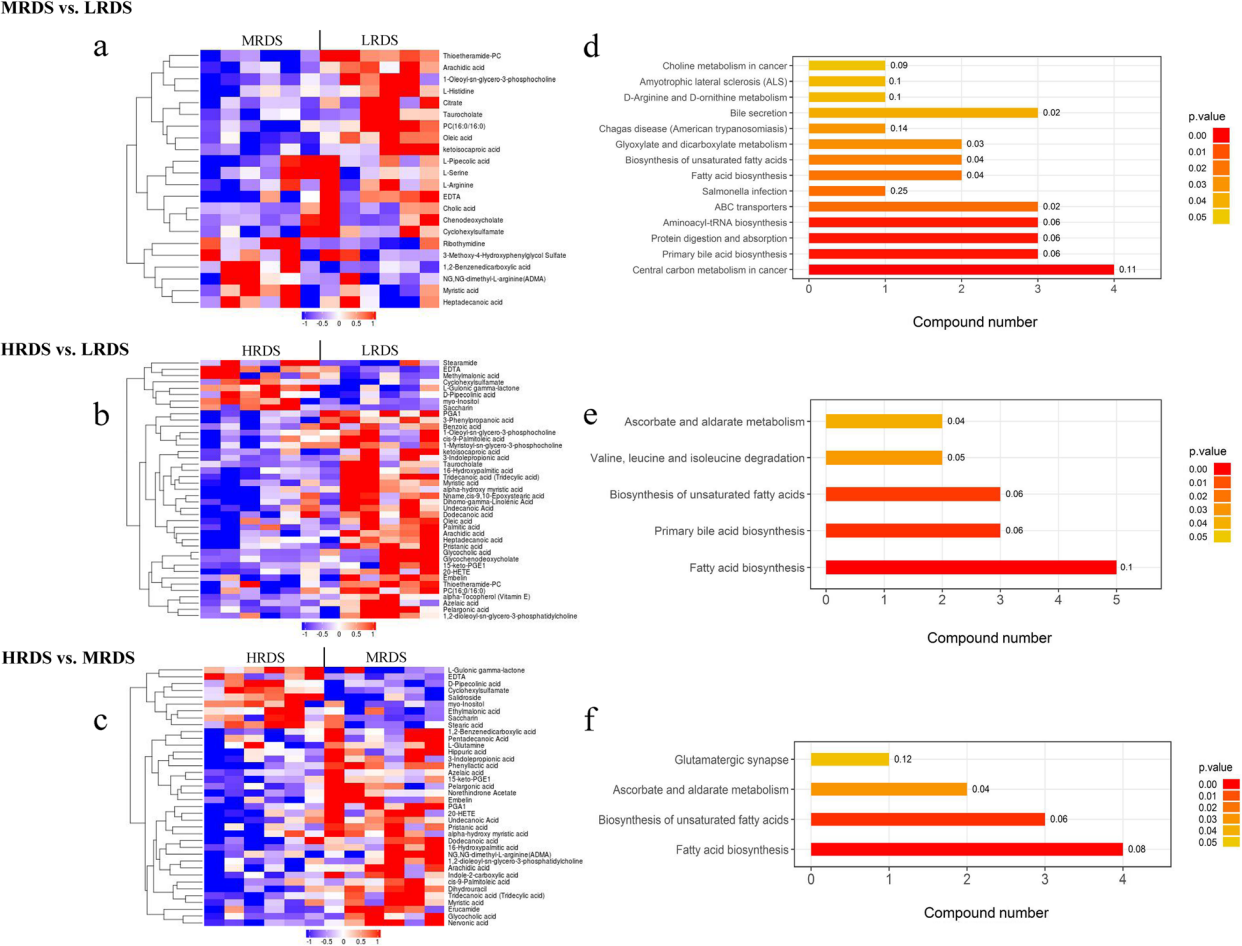
Metabolomic analysis of mammary vein plasma among the three different RDS groups. **Note: a**, **b**, and **c** represent the heat maps of differential metabolites in MRDS vs. LRDS, HRDS vs. LRDS and HRDS vs. MRDS, respectively. The upregulated metabolites are shown in red, whereas the downregulated metabolites are presented in blue. **d**, **e**, and **f** represent the KEGG pathway analysis in MRDS vs. LRDS, HRDS vs. LRDS and HRDS vs. MRDS based on differential metabolites in both positive and negative modes, respectively. The number after the bar represents the enrichment factor

Further metabolomic analysis was performed to study how metabolic alterations influenced the occurrence of MFD. The OPLS-DA score plots were generated for both positive and negative modes, and the values of corresponding R^2^Y and Q^2^ indicated that the predictive capabilities of the models were reliable (Fig. S1). Univariate analysis allowed for the concentration of regulated metabolites in plasma to be compared separately between different RDS groups. The identified differential metabolites (VIP > 1, *P* < 0.1) in the MRDS vs. LRDS, HRDS vs. LRDS and HRDS vs. MRDS group comparisons are shown in Table S5. Heat map analysis revealed significant differences based on the differential metabolites in each compared group (Fig. 1a-c).

In total, 22 differential metabolites were identified between the LRDS and MRDS groups (Fig. 1a). Compared with those in the LRDS group, the metabolites related to lipid metabolism, including chenodeoxycholate, cholic acid, arachidic acid, oleic acid and taurocholate, tended to decrease in the MRDS group (*P* = 0.053–0.092). There were 41 metabolites altered in the HRDS group when compared with those in the LRDS group (Fig. 1b). Of these, the concentrations of arachidic acid, undecanoic acid, tridecanoic acid and myristic acid were significantly reduced (*P* < 0.05) in the HRDS group. The concentrations of palmitic acid, *cis*-9 palmitoleic acid, dihomo-gamma-linolenic acid, pelargonic acid, dodecanoic acid, heptadecanoic acid and oleic acid tended to decrease in the HRDS group compared to those in the LRDS group (*P* = 0.058–0.096). A total of 38 differential metabolites were identified in the HRDS group when compared with those in the MRDS group (Fig. 1c). Of these, 29 metabolites were reduced in the HRDS group. The significant decrease in myristic acid, arachidic acid, stearic acid, and nervonic acid may have been associated with the occurrence of MFD. Moreover, the concentrations of alpha-hydroxy myristic acid, dodecanoic acid, *cis*-9 palmitoleic acid and pentadecanoic acid tended to decrease in the HRDS group compared to those in the MRDS group (*P* = 0.051–0.089). Moreover, KEGG pathway analysis based on differential metabolites again proved that lipid metabolism processes, including the biosynthesis of fatty acids, unsaturated fatty acids, and primary bile acid, were significantly disturbed when the dietary RDS percentage was increased (Fig. 1d-f).

### The decrease in milk fat synthesis was probably due to the downregulation of *INSIG1*

**Fig. 2:**
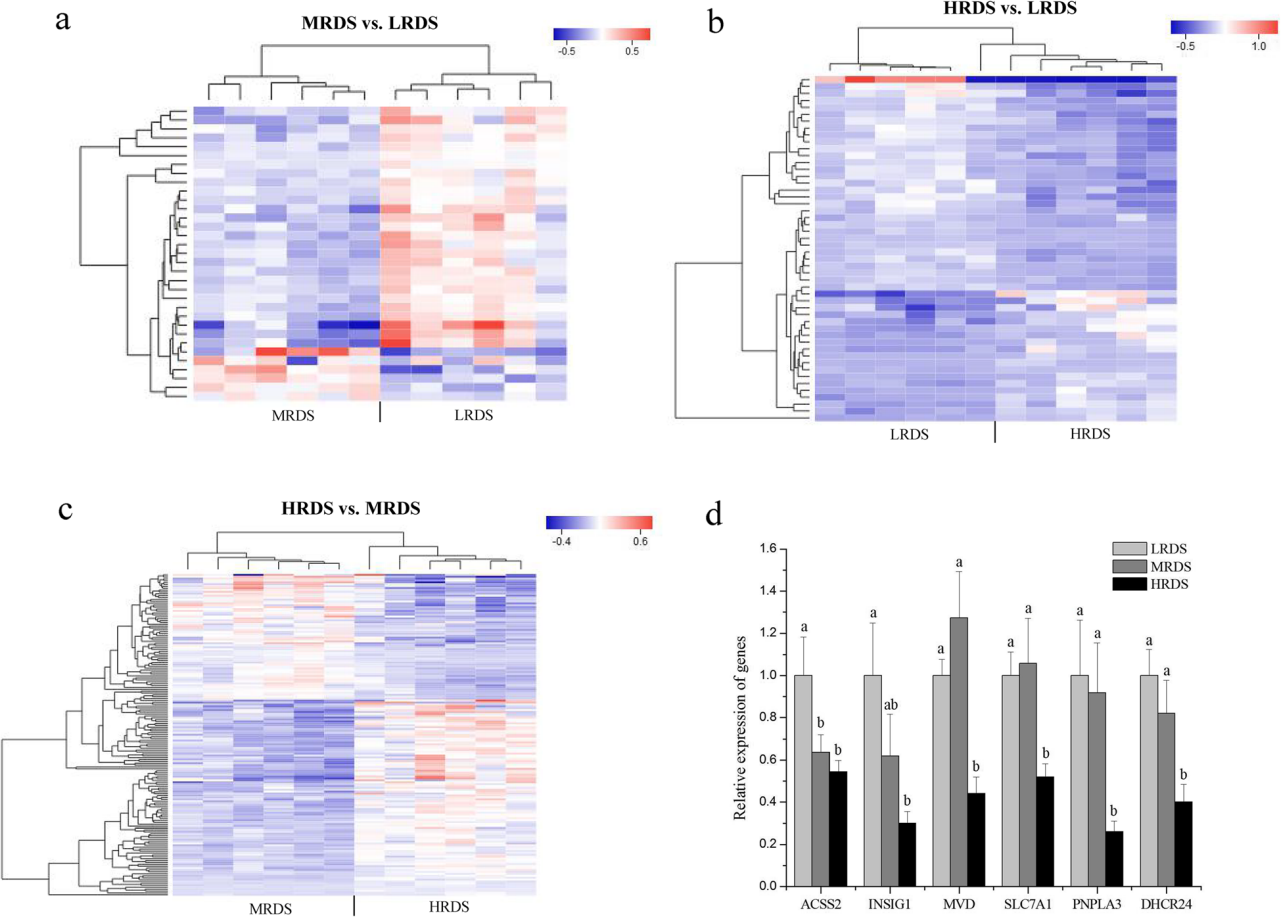
Transcriptomic analysis of mammary tissue among the three different RDS groups. **Note: a**, **b**, and **c** represent the heat maps of differentially expressed genes in MRDS vs. LRDS, HRDS vs. LRDS and HRDS vs. MRDS, respectively. The upregulated metabolites are shown in red, whereas the downregulated metabolites are presented in blue. d represent the results of qPCR validation of RNA-sequencing data

A total of 33, 50, and 169 DEGs were identified in the comparison groups of MRDS vs. LRDS, HRDS vs. LRDS, and HRDS vs. MRDS (Table S6). The heat map revealed a clear bifurcation of MRDS vs. LRDS (Fig. 2a) and HRDS vs. MRDS (Fig. 2c). However, the bifurcation of HRDS vs. LRDS was not very clear (Fig. 2b). The qRT-PCR analyses proved that the transcriptomic analyses were reproducible and reliable (Fig. 2d). To gain a better understanding of the functional roles of these differentially expressed genes among the 3 dietary groups, further GO (Table S7) and KEGG (Table S8) analyses were performed. Most of the top 10 GO terms were related to lipid metabolism, of which only lipid biosynthetic processes were significantly enriched (FDR < 0.05). The significant DEGs, including *ACSS2*, *MVD*, *INSIG1*, *AGPS*, *SCD5*, *FADS2*, *CERCAM*, *SC5D*, *HSD17B7*, *HSD17B12*, *ATM*, *TP53RK*, *GDF1 and LOC102177400,* were involved in the lipid biosynthetic process. Moreover, two changed pathways, namely, those of steroid biosynthesis (FDR = 0.005; 4 genes) and the biosynthesis of unsaturated fatty acids (FDR = 0.007; 4 genes), were significantly enriched (FDR < 0.05) according to the KEGG analysis. The significant DEGs involved in these 2 pathways included *SCD5*, *FADS2*, *DHCR24*, *HSD17B12*, *SC5D*, *LOC102177400* and *ACOT1_2_4* (*LOC102181962*).

**Fig. 3:**
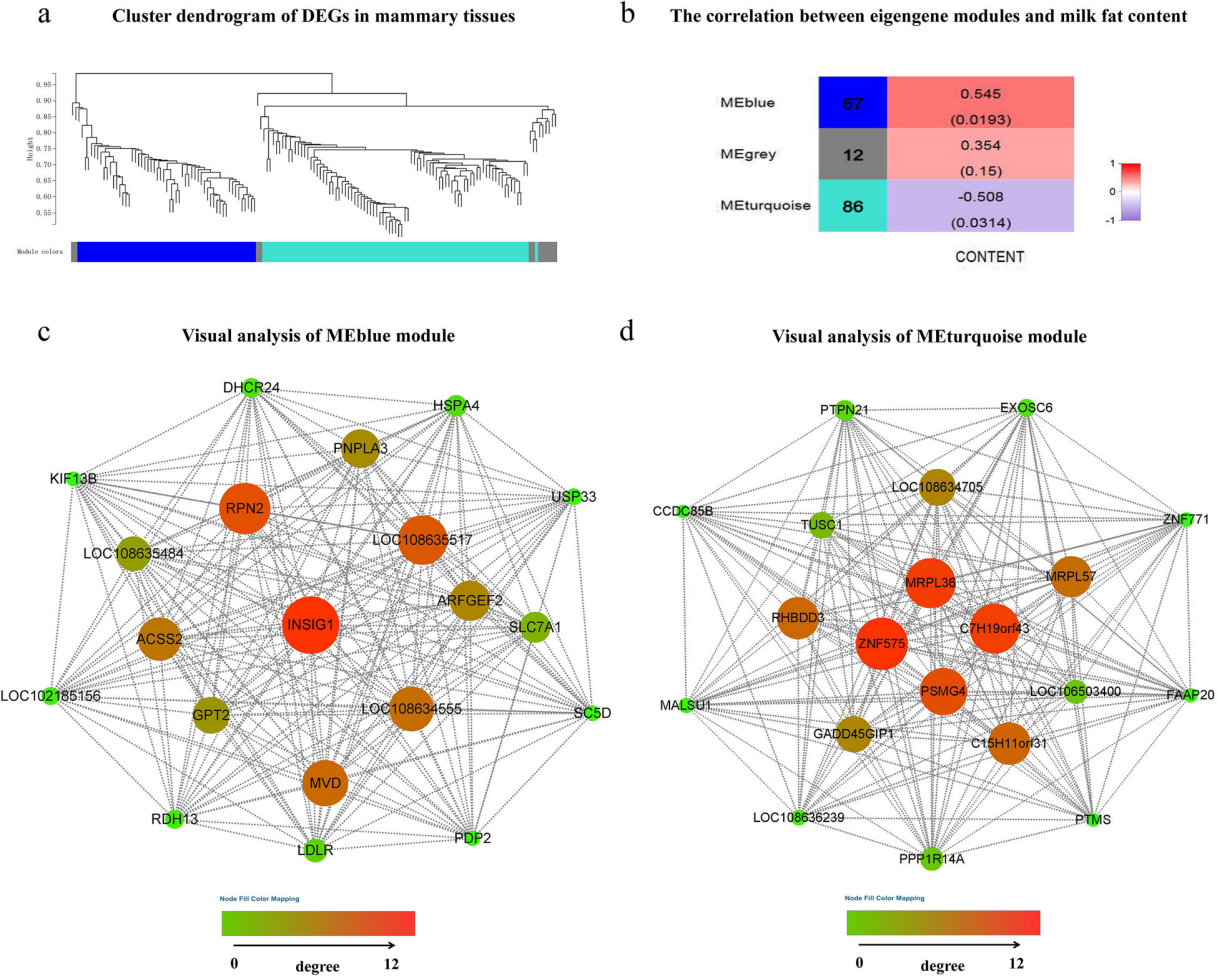
WGCNA of DEGs in the mammary glands of dairy goats. **Note: a** hierarchical cluster tree conducted by WGCNA showing coexpression modules. Each leaf represents a gene, and each module corresponds to branches marked by different colors. **b** The correlation between eigengene modules and milk fat content. Red and purple represent positive and negative correlations, respectively, and the values in parentheses represent significance (*P* < 0.05). **c** Gene coexpression network of the blue module. The size and color of the nodes represent the degree of connectivity of the corresponding genes. **d** Gene coexpression network of the turquoise module. The size and color of the nodes represent the degree of connectivity of the corresponding genes

The interaction between DEGs in the mammary tissue of goats was predicted by the R package “WGCNA”. Three modules were clustered: the blue, grey and turquoise modules (Fig. 3a). Based on the correlation coefficients between module and phenotype, the blue module and turquoise module were significantly positively and negatively correlated with milk fat content, respectively (Fig. 3b). The gene coexpression network of the blue module (Fig. 3c) and turquoise module (Fig. 3d) were visualized by Cytoscape software. Based on the GO database, most of the top 20 nodes of the blue module’s gene coexpression network were associated with lipid metabolism. Among them, *INSIG1* had the highest degree of connectivity. The nodes of the turquoise module’s gene coexpression network were related to nucleic acid binding (*ZNF575*, *ZNF771*), RNA degradation (*EXOSC6*), negative regulation of transcription (*CCDC85B*), negative regulation of catalytic activity (*PPP1R14A*) and negative regulation of mitochondrial translation (*MALSU1*).

## Discussion

MFD can affect milk quality and is currently a topic of great interest for dairy ruminant nutrition research [38]. Compared with cow milk, goat milk contains more CLA, less cholesterol, and smaller milk fat globules, which are beneficial to the cardiovascular health of humans [39]. However, previous studies have mainly focused on the occurrence of MFD in dairy cows, but limited research has focused on the occurrence of MFD in dairy goats, whose milk quality may be influenced more by MFD but remains unclear [40]. Moreover, previous studies mainly focused on the effect of high-concentration diets on the occurrence of MFD, which mainly resulted from rumen fermentation abnormalities [8, 41]. Compared with the dietary starch content, the effects of the RDS on rumen fermentation were even greater and have rarely been studied before.

In this study, the milk fat content and yield in the HRDS group were reduced by 19.5 and 40.1%, respectively, compared with those in the LRDS group. The yield of almost all fatty acids was decreased in the HRDS group compared with those in the LRDS and MRDS groups, the total saturated fatty acids (SFAs) decreased by 43.5% in the HRDS group, and the most affected SFAs were short- and medium-chain fatty acids. Other studies on MFD also confirmed these results [14, 42]. These above results indicated that MFD was identified in the HRDS groups. Moreover, the SCC in the HRDS group increased significantly compared to that in the LRDS and MRDS groups. In a previous study, the area of ruminal pH under 5.8 or 5.6, as well as the duration of pH below 5.8 or 5.6, were significantly increased with high-RDS treatment. The results indicated that an HRDS diet may cause subacute rumen acidosis in goats [22]. This subhealthy state may lead to an increase in SCC. However, based on mammary tissue transcriptomics analysis, we did not observe enriched pathways related to inflammation. The specific relationship between HRDS treatment and SCC needs to be studied further. However, the LRDS and MRDS diets did not induce MFD, although the concentrate-to-fiber ratio and total starch content were the same as those of the HRDS diet. This also means that we can reduce the risk of using high concentrate diets by regulating the amount of RDS.

The results may have been induced by a high dietary RDS content, which could have altered the ruminal biohydrogenation of polyunsaturated fatty acids and increased the production of trans fatty acid intermediates that inhibit milk fat synthesis. In previous studies of dairy cows, excessive starch caused a decrease in rumen pH, which, in turn, led to a decrease in the relative abundance of rumen hydrogenated bacteria and increased the content of milk fat inhibitors (such as *trans*-10, * cis*-12 CLA) [14, 43]. *Trans*-10, * cis*-12 CLA is produced in the rumen and transmitted through the blood to the mammary glands, where it impairs the production of several enzymes involved in milk fat synthesis [42, 44]. In the rumen, CLA isomers are intermediate in the biohydrogenation of linoleic acid to stearic acid. In the normal biohydrogenation pathway, C18:2n-6 is first converted to *cis*-9, * trans*-11 CLA, then converted to *trans*-11 C18:1, and, finally, converted to C18:0. However, this normal pathway could be transformed into an alternate pathway due to high-RDS sources or the lack of effective fiber, finally producing *trans*-10 C18:1 and the potent milk fat inhibitor *trans*-10, * cis*-12 CLA [9, 45, 46]. Similar to previous studies, the concentrations of *trans*-10, * cis*-12 CLA and *trans*-10 C18:1 in the rumen were significantly higher in the HRDS group than in the other two groups in our study. Although further study is needed on whether *trans*-10 C18:1 can directly cause MFD, the concentration of *trans*-10 C18:1 was negatively correlated with milk fat yield, and it is commonly used as a proxy for the persistence of altered rumen biohydrogenation pathways [9].

Some studies reported that accumulation of *trans*-C18:1 fatty acids in the rumen and flow of *trans*-C18:1 at the omasum were associated with changes in the relative abundance of certain strains of *Butyrivibrio* [9, 47, 48]. Based on the determination of the relative abundance of rumen eubacteria, different levels of RDS did not cause significant changes in hydrogenation-involved bacteria except for *B. fibrisolvens + Pseudobutyrvibrio*, which are important type A hydrogenated hydrogenating bacteria that can reduce C18:2n-6 and C18:3n-3 to *trans*-11 C18:1 [9, 49, 50]. The decreased abundance of *B. fibrisolvens + Pseudobutyrvibrio* in the HRDS group resulted in a hindrance to the process of converting C18:2n-6 to *trans*-11 C18:1, which, in turn, caused more C18:2n-6 to be converted to *trans*-10, * cis*-12 CLA, ultimately increasing the *trans*-10, * cis*-12 CLA content in the rumen. Hence, our study indicated that *B. fibrisolvens + Pseudobutyrvibrio* played important roles in producing *trans*-10, * cis*-12 CLA and inhibiting milk fat synthesis when the RDS was increased in the diets of dairy goats.

To better understand the effect of RDS on milk fat synthesis, focus should not only be on the metabolism of the rumen but also on the lipid metabolism of the mammary tissue. Pathway analysis from metabolomics has shown that lipid metabolism is disturbed by an HRDS diet [51]. Bile acids act as signaling molecules that coordinately regulate lipid, glucose, and other energy metabolism pathways [52]. As the milk fat decreased, the concentration of primary bile acids also changed in our study. The enriched primary bile acid biosynthesis pathway was accompanied by multiply decreased glycochenodeoxycholate, chenodeoxycholate, glycocholic acid, taurocholate and cholic acids in the HRDS and MRDS groups compared with the those in the LRDS group. Considering that bile acids are derived from cholesterol [53], we supposed that the primary bile acid biosynthesis pathway was weakened due to the downregulation of genes associated with cholesterol and steroid biosynthesis. This hypothesis was verified by transcriptomic analysis of the mammary tissue.

Our RNA-Seq analysis showed that some key genes associated with lipid biosynthesis were downregulated in the HRDS group. In the process of *de novo* fatty acid synthesis, it is necessary to activate acetate to acetyl-CoA through the action of ACSS2, thereby becoming a substrate for milk fat synthesis [54]. Our study revealed that the mRNA abundance of *ACSS2* was significantly decreased with HRDS treatment. Moreover, cholesterol and steroid biosynthesis-related pathways were significantly depressed in the mammary tissue of goats fed the HRDS diet. Of these pathways, 5 key genes involved in these pathways, including *MVD*, *HSD17B7*, *LOC102177400*, *LDLR*, and *INSIG1*, were found to be significantly downregulated in the HRDS group. These results indicated that the downregulation of these genes involved in fatty acid and cholesterol synthesis processes could result in the depression of milk fat synthesis.

**Fig. 4:**
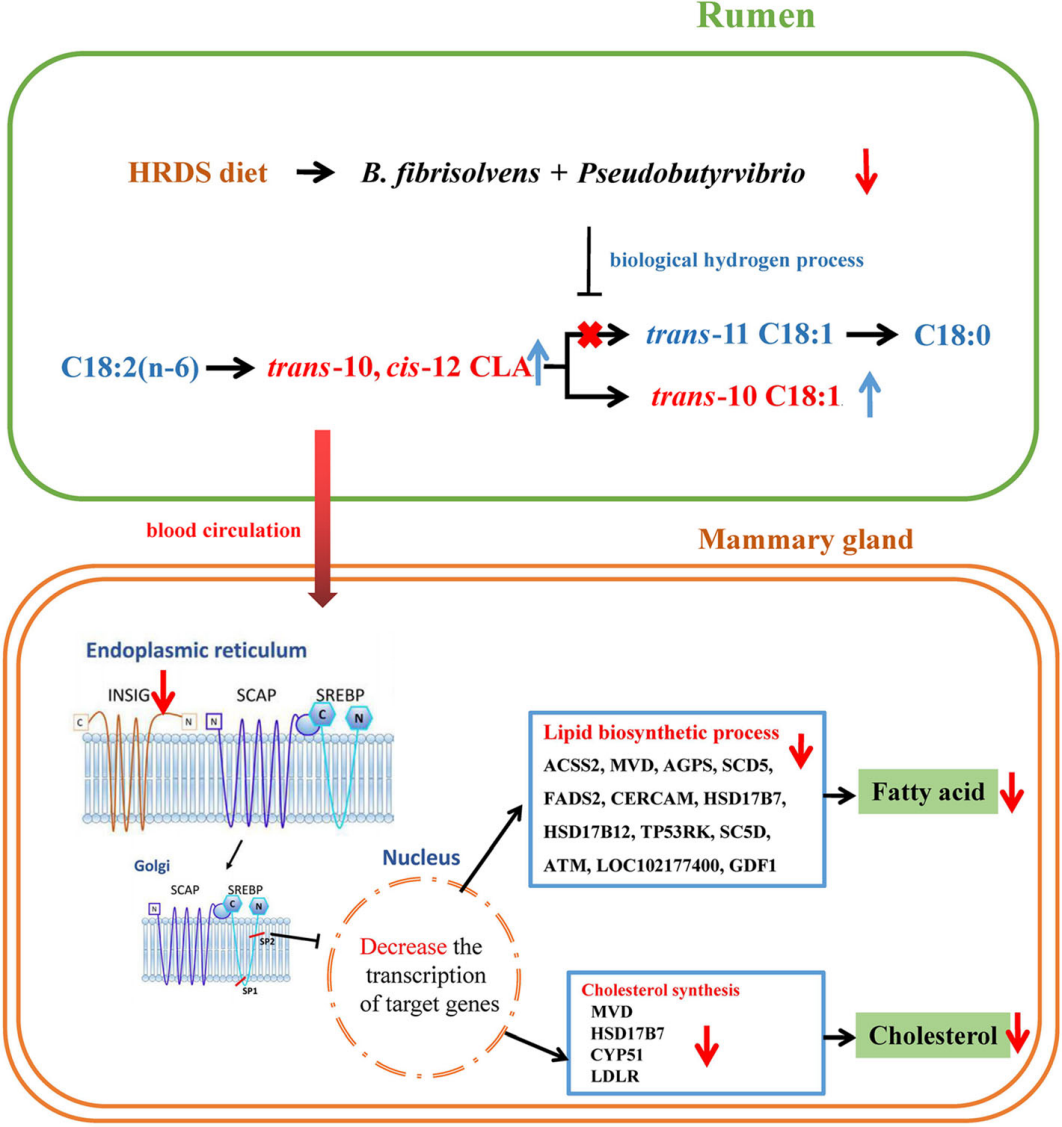
Interrelationships among proteins codified by genes within downregulated pathways and GO terms in HRDS treatment. **Note:** Red arrows denote downregulation. The blue box contains significantly downregulated genes (black) and their function (red). The green box represents products of the proteins codified by the down-regulated genes

Research has demonstrated that the family of transcription factors *SREBF1* is highly expressed in bovine mammary tissue and activates genes that regulate FA and triglyceride biosynthesis [55]. Moreover, Horton et al. [56] reported that the expression of cholesterol synthesis genes was also regulated by *SREBF1*. Hence, the depression of milk fat synthesis could be regulated by the activity of SREBP. A study by Harvatine and Bauman [57] showed significant downregulation of *SREBF1, INSIG1, THRSP* and mature SREBP proteins in bovine mammary gland tissues of cows by jugular vein perfusion of *trans*-10, * cis*-12 CLA (10 g/d). In our study, there was no significant difference in the expression of *SREBF1*. However, *INSIG1* was significantly downregulated in the HRDS treatment group. According to the results of WGCNA, *INSIG1* was the hub gene with the highest degree of connectivity and interacted with multiple genes involved in lipid metabolism. These results indicated that *INSIG1* could be a hub gene, which indicated that a decrease in *INSIG1* expression may reduce the activity of SREBP and then downregulate the expression of genes downstream of *SREBF1*. The synthesis of fatty acids and cholesterol is regulated by the INSIG-SCAP-SREBP complex. SREBP is associated with SCAP and forms a SREBP-SCAP complex that is retained in the ER by binding to INSIG when inactive. Activation is achieved by dissociating INSIG, which allows for translocation to the Golgi. Hence, *INSIG1* is known as a key component of homeostatic regulation by controlling the activity of SREBP [58]. Hence, the inhibition of fatty acid synthesis was likely due to a decrease in SREBP activity caused by downregulation of *INSIG1,* although there was no difference in the mRNA abundance of *SREBF1*. Moreover, previous studies demonstrated that endoplasmic reticulum stress and apoptosis could be induced by *trans*-10, * cis*-12 CLA [59–61], while endoplasmic reticulum stress could further lead to the depletion of *INSIG1*, which is in accordance with the results of the present study. Moreover, we found that some genes were significantly upregulated as the milk fat content decreased. The results indicated that the occurrence of MFD may be related to the biological process of negative transcription regulation (*CCDC85B*), negative mitochondrial regulation (*MALSU1*), negative catalytic activity regulation (*PPP1R14A*) and the molecular function of nucleic acid binding (*ZNF575*, *ZNF771*). The specific relationship between upregulated genes and MFD needs to be studied further. A summary of the principal findings regarding the downregulated genes of the goat mammary glands in the HRDS group is presented in Fig. 4. It is worth mentioning that the low, medium and high-RDS levels may not be linear in energy supply to mammary gland tissue. Rumen starch digestion usually accounts for 75–80% of starch intake. Additionally, 35–60% of the starch entering the small intestine is degraded, and 35–50% of the portion that escapes digestion in the small intestine is degraded in the large intestine. However, the digestibility of starch in the large intestine is low, causing a huge loss of its postruminal digestive efficiency [62]. In our study, the three diets with the same total starch content but different RDS contents also contained different intestinal degradable starch (IDS) contents. The LRDS diet has a higher IDS content than that of the MRDS and HRDS diets, which may increase the energy loss caused by hindgut fermentation. However, the RDS level in the HRDS diet may be too high and cause MFD. Based on the analysis of milk yield, lactation efficiency (FPCM/DMI) and milk fat yield, the ratio of RDS to IDS in the MRDS diet may be the most appropriate of the three treatments. This may be why the heat map bifurcation of differentially expressed genes in HRDS vs. LRDS was unclear (Fig. 2b), although the key genes associated with milk fat synthesis were significantly reduced in the HRDS group compared to those in the LRDS group.

## Conclusion

In summary, low RDS and medium RDS diets did not cause MDF in lactating goats, even if the total starch content and the concentrate-to-fiber ratio were high. Increased dietary RDS levels in goat feedstuff could decrease the abundance of rumen *B. fibrisolvens* + *Pseudobutyrvibrio* and result in the downregulation of the mammary *INSIG1* gene, thereby reducing *de novo* fatty acid synthesis and milk fat production and leading to MFD in lactating goats, which was determined by systematically studying the occurrence of MFD induced by increased RDS levels. Moreover, as the level of RDS increased, cholesterol and primary bile acid biosynthetic processes were also inhibited, which could also serve as important features of MFD in the future. In summary, our results suggested that a high dietary RDS content has a negative impact on milk fat synthesis in dairy goats, which helps us better understand the mechanisms of increased RDS on milk fat synthesis and how to adjust the feeding strategy to use starch-rich diets more effectively.

## Supplementary information

**Additional file 1: Table S1.** Ingredients and chemical composition of diets. **Table S2.** The specific primers for the qRT-PCR of *GAPDH* and the tested mRNAs. **Table S3.** The specific primers used for qRT-PCR of eubacteria. **Table S4.** Concentration of major fatty acids in the different RDS diets. **Table S5.** Identified differential metabolites in MRDS vs. LRDS, HRDS vs. LRDS and HRDS vs. MRDS. **Table S6.** The differentially expressed genes in the compared groups of MRDS vs. LRDS, HRDS vs. LRDS and HRDS vs. MRDS. **Table S7.** Gene Ontology analysis of differentially expressed genes among the treatments. **Table S8.** KEGG pathway analysis of differentially expressed genes among the treatments. **Figure S1.** OPLS-DA score of MRDS vs. LRDS, HRDS vs. LRDS and HRDS vs. MRDS in positive mode and negative mode. Note: **a**, **b**, and **c** represent the OPLS-DA scores of MRDS vs. LRDS, HRDS vs. LRDS and HRDS vs. MRDS in positive mode, respectively. **d**, **e**, and **f** represent the OPLS-DA scores of MRDS vs. LRDS, HRDS vs. LRDS and HRDS vs. MRDS in negative mode, respectively.

## Data Availability

The authors confirm that all data underlying the findings are fully available without restriction.

## Abbreviations

CLA: Conjugated linoleic acid
*INSIG1*: Insulin induced gene 1
MFD: Milk fat depression
qRT-PCR: Quantitative real-time PCR
RDS: Rumen-degradable starch
RES: Rumen escaped starch
SREBF1: Sterol regulatory element binding transcription factor 1
SREBP: Sterol regulatory element binding protein
