# Supplementary material for: High rumen degradable starch decreased goat milk fat via trans-10, cis-12 conjugated linoleic acid-mediated downregulation of lipogenesis genes, particularly, INSIG1
Source: J Anim Sci Biotechnol. 2020 Apr 6;11:30. doi: 10.1186/s40104-020-00436-3 (PMC7132897; doi:10.1186/s40104-020-00436-3)

**Title:** High rumen degradable starch decreased goat milk fat via *trans*-10,*cis*-12 conjugated linoleic acid-mediated downregulation of lipogenesis genes, particularly, *INSIG1*

**Authors:** Lixin Zheng^*, ¶^, Shengru Wu^*, ¶^, Jing Shen^*^, Xiaoying Han^*^, ChunJia Jin^*^, XiaoDong Chen^*^, Junhu Yao^*^, **^†^**

**Institution:** ^*^ College of Animal Science and Technology, Northwest A&F University, Yangling 712100, Shaanxi, China

^¶^ co-first author: these 2 authors contributed equally to this work

**^†^Corresponding authors:**

JunhuYao, email: yaojunhu2004@sohu.com (J. H. Yao); Tel.: +86 13891883031.

**Supplementary Information**

Table S1 Ingredients and chemical composition of diets.

Table S2 The specific primers for the qRT-PCR of *GAPDH* and tested mRNAs.

Table S3 The specific Primers Used for qRT-PCR of eubacteria.

Table S4 Concentration of major fatty acids of the diets with three different RDS levels.

Table S5 Identified differential metabolites in MRDS vs. LRDS, HRDS vs. LRDS and HRDS vs. MRDS.

Table S6 The differentially expressed genes in the compared groups of MRDS vs. LRDS, HRDS vs. LRDS and HRDS vs. MRDS.

Table S7 Gene Ontology analysis of differentially expressed genes among there treatments.

Table S8 KEGG pathways analysis of differentially expressed genes among there treatments.

Figure S1 OPLS-DA score of MRDS vs. LRDS, HRDS vs. LRDS and HRDS vs. MRDS in the positive mode and negative mode.

**Table S1** **Ingredients and chemical composition of diets**

| Item | Treatment^1^ | | |
| --- | --- | --- | --- |
|  | L-RDS | M-RDS | H-RDS |
| Ingredient, g/kg of DM |  |  |  |
| Alfalfa hay | 17.50 | 17.50 | 17.50 |
| Corn silage | 27.50 | 27.50 | 27.50 |
| Corn | 40.00 | 23.50 | 8.00 |
| Wheat | - | 18.00 | 36.00 |
| Soybean meal | 13.00 | 7.60 | 5.00 |
| Corn gluten meal | - | 1.90 | 2.00 |
| Wheat bran | - | 2.00 | 2.00 |
| Calcium phosphate | 0.25 | 0.25 | 0.25 |
| Limestone | 0.75 | 0.75 | 0.75 |
| Salt | 0.50 | 0.50 | 0.50 |
| Vitamin-mineral mix | 0.50 | 0.50 | 0.50 |
| Nutrient composition, g/kg of DM, unless noted | | | |
| DM, g/kg of fresh | 50.58 | 50.27 | 50.21 |
| ADF | 18.88 | 18.58 | 18.22 |
| NDF | 34.38 | 33.76 | 32.90 |
| CP | 16.40 | 16.71 | 16.59 |
| Starch | 27.66 | 27.54 | 28.58 |
| RDS | 20.52 | 22.15 | 24.88 |
| NE_L_^2^, MJ/kg | 7.14 | 7.11 | 7.11 |

^1^Treatment were LRDS diet (RDS=20.52%), MRDS diet (RDS=22.15%), and HRDS diet (RDS=24.88%) with similar total starch content.

^2^NE_L_ = Net energy for lactation

**Table S2 The specific primers for the qRT-PCR of *GAPDH* and tested mRNAs**

| Gene | Accession number | Primer sequence (5′ → 3′) | Product size, bp |
| --- | --- | --- | --- |
| *ACSS2* | XM_018057751.1 | F: GGCGAATGCCTCTACTGCTT | 100 |
|  |  | R: GGCCAATCTTTTCTCTAATCTGCTT |  |
| *INSIG1* | NM_001286088.1 | F: TGCTGGTGTACAACGGAGTC | 281 |
|  |  | R: GTCACTGGTACAGTCAGCCC |  |
| *MVD* | XM_018061849.1 | F: AGCGATCTGTCAGAAGTGGC | 174 |
|  |  | R: ACTCACCACGAGGATCAGGA |  |
| *SLC7A1* | XM_018056608.1 | F: CCCGCCCTCCACACTTCATT | 214 |
|  |  | R: GACTGGACTGATGGACGCTC |  |
| *PNPLA3* | XM_005709739.3 | F: CTCGCTTACCAGAGTGTCCG | 88 |
|  |  | R: GGAACAATACAGGGCGTCCA |  |
| *DHCR24* | XM_005678362.3 | F: CGAGATAAGGCCCCATGCTT | 96 |
|  |  | R:GGACTGAGGACAACAGAGGC |  |
| *GAPDH* | XM_005680968.3 | F:TGGAGAAACCTGCCAAGTATGA | 138 |
|  |  | R: AGGTAGAAGAGTGAGTGTCGC |  |
| *UXT* | XM_005700842.2 | F: CAGCTGGCCAAATACCTTCAA | 125 |
|  |  | R: GTGTCTGGGACCACTGTGTCAA |  |
| *MRPL39* | XM_005674737.3 | F: AGCTGTCACTAACTCCCCGA | 71 |
|  |  | R:AGCTGTCACTAACTCCCCGA |  |

**Table S3 The specific Primers Used for qRT-PCR of eubacteria.**

| Target organisms | Primer | Primer sequence (5′ → 3′) | Reference |
| --- | --- | --- | --- |
| Total eubacteria | Forward | GTGSTGCAYGGYTGTCGTCA | [1] |
|  | Reverse | ACGTCRTCCMCACCTTCCTC |  |
| *B. proteoclasticus* | Forward | TCCGGTGGTATGAGATGGGC | [2] |
|  | Reverse | GTCGCTGCATCAGAGTTTCCT |  |
| *B. fibrisolvens* +*Pseudobutyrivibrio* spp. | Forward | GCCTCAGCGTCAGTAATCG | [3] |
|  | Reverse | GGAGCGTAGGCGGTTTTAC |  |
| *B. hungatei* | Forward | AGGGTAATGCCTGTAGCTC | [3] |
|  | Reverse | TCACCCTCGCGGGAT |  |
| Atypical *B. fibrisolvens* | Forward | GACGGTGTATCAAGTCTGAAGTG | [3] |
|  | Reverse | GCCGGCACTGAAAGACTATGTC |  |
| *P. acnes* | Forward | F:GGGTTGTAAACCGCTTTCGCCTG | [3] |
|  | Reverse | R:TGCTTTCGATACGGGTTGAC |  |
| *S. bovis* | Forward | F:ATGTTAGATGCTTGAAAGGAGCAA | [4] |
|  | Reverse | R:CGCCTTGGTGAGCCGTTA |  |

**Supplemental Tables3 literature cited**

[1] Maeda H, Fujimoto C, Haruki Y, Maeda T, Kokeguchi S, Petelin M, et al. Quantitative real-time PCR using TaqMan and SYBR Green for *Actinobacillus actinomycetemcomitans*, *Porphyromonas gingivalis*, *Prevotella* *intermedia*, *tet*Q gene and total bacteria. FEMS Immunol Med Microbiol 2003; 39:81-6.

[2] Paillard D, McKain N, Rincon MT, Shingfield KJ, Givens DI, Wallace RJ. Quantification of ruminal *Clostridium proteoclasticum* by real-time PCR using a molecular beacon approach. J Appl Microbiol 2007;103:1251–61.

[3] Shingfield KJ, Kairenius P, Arola A, Paillard D, Muetzel S, Ahvenjärvi S, et al. Dietary fish oil supplements modify ruminal biohydrogenation, alter the flow of fatty acids at the omasum, and induce changes in the ruminal *Butyrivibrio* population in lactating cows. J Nutr. 2012; 142(8): 1437-1448.

[4] Klieve AV, Hennessy D, Ouwerkerk D, Forster RJ, Mackie RI, Attwood GT. Establishing populations of *Megasphaera elsdenii* YE 34 and *Butyrivibrio fibrisolvens* YE 44 in the rumen of cattle fed high grain diets. J Appl Microbiol. 2003;95:621-30.

**Table S4** **Concentration of major fatty acids of the diets with three different RDS levels.**

| FA concentration,  g/100 g of total FA | Treatment^1^ | | | SEM | *P-value* |
| --- | --- | --- | --- | --- | --- |
|  | LRDS | MRDS | HRDS |  |  |
| C16:0 | 21.21 | 21.24 | 23.5 | 0.46 | 0.113 |
| *cis*-9 C16:1 | 0.26 | 0.27 | 0.29 | 0.01 | 0.307 |
| C18:0 | 3.47 | 3.32 | 3.13 | 0.08 | 0.225 |
| *cis*-9 C18:1 | 22.66^a^ | 21.19^b^ | 18.89^c^ | 0.51 | ＜0.001 |
| C18:2n-6 | 44.16 | 43.62 | 44.93 | 0.59 | 0.701 |
| C18:3n-3 | 0.21 | 0.13 | 0.15 | 0.02 | 0.354 |
| C18:3n-6 | 0.10 | 0.11 | 0.09 | 0.01 | 0.065 |
| C20:0 | 4.77^b^ | 5.47^a^ | 5.70^a^ | 0.15 | 0.007 |
| *cis*-9 C20:1 | 0.35^b^ | 0.48^a^ | 0.53^a^ | 0.03 | 0.002 |
| SFA^2^ | 31.70 | 33.41 | 34.86 | 0.66 | 0.149 |
| MUFA^3^ | 23.26^a^ | 21.97^a^ | 19.72^b^ | 0.49 | 0.001 |
| PUFA^4^ | 45.00 | 44.53 | 45.73 | 0.56 | 0.719 |

^a-c^ Means within same row with different superscripts differ (*P*< 0.05).

^1^Treatment were LRDS diet (RDS=20.52%), MRDS diet (RDS=22.15%), and HRDS diet (RDS=24.88%) with similar total starch content.

^2^SFA = saturated fatty acid.

^3^MUFA = monounsaturated fatty acid.

^4^PUFA = polyunsaturated fatty acid.

**Table S5 Identified differential metabolites in MRDS vs. LRDS, HRDS vs. LRDS and HRDS vs. MRDS.**

| Metabolites | VIP^1^ | FC^2^ | *P*-value |
| --- | --- | --- | --- |
| **MRDS vs. LRDS** | 2.828 | 0.344 | 0.001 |
| EDTA | 3.729 | 0.617 | 0.003 |
| PC(16:0/16:0)^3^ | 1.942 | 1.466 | 0.029 |
| Myristic acid | 1.135 | 0.734 | 0.029 |
| *L*-Histidine | 1.378 | 1.382 | 0.029 |
| 1,2-Benzenedicarboxylic acid | 1.054 | 0.687 | 0.031 |
| Cyclohexylsulfamate | 4.228 | 0.568 | 0.037 |
| Citrate | 1.583 | 1.303 | 0.046 |
| 3-Methoxy-4-hydroxyphenylglycol sulfate | 1.941 | 1.779 | 0.051 |
| NG,NG-dimethyl-L-arginine(ADMA) | 1.353 | 0.368 | 0.053 |
| Chenodeoxycholate | 1.243 | 1.440 | 0.054 |
| Heptadecanoic acid | 1.356 | 0.603 | 0.056 |
| *L*-Pipecolic acid | 1.335 | 1.678 | 0.056 |
| Ribothymidine | 1.841 | 0.456 | 0.058 |
| Ketoisocaproic acid | 3.178 | 0.704 | 0.070 |
| *L*-Arginine | 2.386 | 0.357 | 0.070 |
| Cholic acid | 1.923 | 0.728 | 0.073 |
| Thioetheramide-PC | 2.494 | 0.657 | 0.077 |
| Arachidic acid | 1.095 | 0.687 | 0.083 |
| *L*-Serine | 2.610 | 0.587 | 0.092 |
| Oleic acid | 3.139 | 0.448 | 0.092 |
| Taurocholate | 6.046 | 0.687 | 0.097 |
| 1-Oleoyl-sn-glycero-3-phosphocholine | 2.828 | 0.344 | 0.001 |
| **HRDS vs. LRDS** |  |  |  |
| Arachidic acid | 3.376 | 0.285 | 0.001 |
| PGA1^4^ | 1.161 | 0.419 | 0.001 |
| 3-Phenylpropanoic acid | 7.415 | 0.266 | 0.002 |
| Tridecanoic acid (Tridecylic acid) | 2.002 | 0.445 | 0.003 |
| *L*-Gulonic gamma-lactone | 7.586 | 1.872 | 0.005 |
| Alpha-Hydroxy myristic acid | 1.192 | 0.579 | 0.008 |
| Myristic acid | 4.771 | 0.555 | 0.014 |
| Pristanic acid | 1.301 | 0.563 | 0.016 |
| Saccharin | 1.319 | 2.045 | 0.021 |
| Glycocholic acid | 2.960 | 0.134 | 0.022 |
| *D*-Pipecolinic acid | 1.760 | 2.732 | 0.025 |
| PC(16:0/16:0)^3^ | 3.411 | 0.602 | 0.026 |
| Undecanoic Acid | 1.144 | 0.612 | 0.032 |
| Azelaic acid | 1.823 | 0.392 | 0.034 |
| 3-Indolepropionic acid | 3.917 | 0.222 | 0.034 |
| myo-Inositol | 1.192 | 1.653 | 0.036 |
| Thioetheramide-PC | 1.422 | 0.752 | 0.043 |
| 20-HETE | 1.210 | 0.653 | 0.050 |
| 16-Hydroxypalmitic acid | 1.311 | 0.504 | 0.056 |
| Palmitic acid | 1.413 | 0.491 | 0.058 |
| Ketoisocaproic acid | 1.604 | 0.430 | 0.058 |
| Glycochenodeoxycholate | 1.521 | 0.185 | 0.059 |
| 1-Oleoyl-sn-glycero-3-phosphocholine | 6.679 | 0.627 | 0.061 |
| EDTA | 27.186 | 2.026 | 0.062 |
| 1-Myristoyl-sn-glycero-3-phosphocholine | 1.940 | 0.619 | 0.063 |
| *cis*-9-Palmitoleic acid | 1.091 | 0.513 | 0.064 |
| Benzoic acid | 1.521 | 0.732 | 0.066 |
| Alpha-Tocopherol (Vitamin E) | 1.613 | 0.418 | 0.066 |
| Dihomo-gamma-Linolenic Acid | 3.071 | 0.556 | 0.066 |
| Pelargonic acid | 3.018 | 0.674 | 0.067 |
| Cyclohexylsulfamate | 1.092 | 1.469 | 0.067 |
| Dodecanoic acid | 2.606 | 0.669 | 0.068 |
| *cis*-9,10-Epoxystearic acid | 1.265 | 0.532 | 0.069 |
| Taurocholate | 3.185 | 0.423 | 0.071 |
| Stearamide | 1.450 | 1.632 | 0.071 |
| Heptadecanoic acid | 3.168 | 0.660 | 0.079 |
| 1,2-Dioleoyl-sn-glycero-3-phosphatidylcholine | 4.159 | 0.618 | 0.080 |
| Embelin | 1.852 | 0.691 | 0.085 |
| 15-Keto-PGE1 | 1.863 | 0.586 | 0.095 |
| Oleic acid | 9.331 | 0.698 | 0.096 |
| Methylmalonic acid | 1.511 | 1.683 | 0.097 |
| **HRDS vs. MRDS** | 1.151 | 0.341 | 0.001 |
| Phenyllactic acid | 1.311 | 0.575 | 0.001 |
| Dihydrouracil | 1.053 | 3.992 | 0.002 |
| Salidroside | 1.319 | 0.460 | 0.003 |
| PGA1^4^ | 1.676 | 2.138 | 0.006 |
| Cyclohexylsulfamate | 1.060 | 0.268 | 0.007 |
| Nervonic acid | 1.536 | 0.461 | 0.007 |
| 16-Hydroxypalmitic acid | 1.654 | 0.565 | 0.012 |
| Undecanoic Acid | 1.421 | 0.572 | 0.014 |
| Norethindrone Acetate | 1.955 | 3.219 | 0.015 |
| *D*-Pipecolinic acid | 1.877 | 0.530 | 0.016 |
| Tridecanoic acid (Tridecylic acid) | 1.229 | 0.660 | 0.018 |
| Pristanic acid | 2.247 | 0.797 | 0.020 |
| Erucamide | 2.608 | 0.433 | 0.022 |
| Arachidic acid | 1.310 | 0.709 | 0.024 |
| 1,2-Benzenedicarboxylic acid | 3.500 | 1.435 | 0.025 |
| Stearic acid | 1.009 | 0.420 | 0.034 |
| Indole-2-carboxylic acid | 5.741 | 1.543 | 0.035 |
| *L*-Gulonic gamma-lactone | 2.657 | 0.642 | 0.035 |
| Embelin | 2.014 | 0.313 | 0.036 |
| Glycocholic acid | 1.794 | 0.651 | 0.039 |
| Hippuric acid | 3.321 | 0.369 | 0.044 |
| 3-Indolepropionic acid | 2.446 | 0.595 | 0.047 |
| 15-Keto-PGE1 | 4.369 | 0.642 | 0.049 |
| Myristic acid | 3.060 | 2.051 | 0.050 |
| EDTA | 1.071 | 0.715 | 0.051 |
| Alpha-Hydroxy myristic acid | 1.066 | 1.470 | 0.052 |
| Ethylmalonic acid | 3.509 | 0.629 | 0.055 |
| Dodecanoic acid | 3.231 | 0.695 | 0.059 |
| Pelargonic acid | 1.185 | 0.737 | 0.067 |
| 20-HETE | 1.282 | 1.566 | 0.069 |
| myo-Inositol | 1.352 | 0.458 | 0.069 |
| *cis*-9-Palmitoleic acid | 1.032 | 0.650 | 0.082 |
| 1,2-Dioleoyl-sn-glycero-3-phosphatidylcholine | 1.233 | 1.658 | 0.088 |
| Saccharin | 1.217 | 0.777 | 0.089 |
| Pentadecanoic Acid | 1.728 | 0.593 | 0.091 |
| NG,NG-dimethyl-*L*-arginine(ADMA) | 1.572 | 0.512 | 0.093 |
| Azelaic acid | 1.393 | 0.626 | 0.094 |
| *L*-Glutamine | 1.151 | 0.341 | 0.001 |

^1^VIP means variable importance in projection.

^2^FC means fold change, calculated as the mean value of the peak area obtained from the treatment group/mean value of the peak area obtained from the control group.

^3^PC (16:00/16:00) means phosphatidylcholine (16:00/16:00).

^4^PGA1 means prostaglandin A1.

**Table S6 The differentially expressed genes in the compared groups of MRDS vs. LRDS, HRDS vs. LRDS, and HRDS vs. MRDS.**

| Gene ID | Gene namee | Fold change/ | Log_2_FC | *P*-value | FDR-value^1^ | Significant | Regulate |
| --- | --- | --- | --- | --- | --- | --- | --- |
| **MRDS vs. LRDS** | | | | | | | |
| gene13427 | *SLCO4A1* | 1.613 | 0.6898 | 0.000139 | 0.06613 | yes | up |
| gene12699 | *MRPL57* | 0.579 | -0.7887 | 0.00002464 | 0.03093 | yes | down |
| gene20523 | *MRPL36* | 0.591 | -0.7598 | 0.00002716 | 0.03093 | yes | down |
| gene12480 | *LRRC26* | 0.614 | -0.7036 | 0.00002815 | 0.03093 | yes | down |
| gene7042 | *MFSD10* | 0.606 | -0.7237 | 7.158E-11 | 0.000001158 | yes | down |
| gene27692 | *CLDN5* | 0.621 | -0.687 | 0.0002259 | 0.09373 | yes | down |
| gene13217 | *THBD* | 1.574 | 0.654 | 0.00009438 | 0.05265 | yes | up |
| gene18000 | *ZNF575* | 0.608 | -0.717 | 0.0001077 | 0.05808 | yes | down |
| gene8530 | *GDF1* | 0.646 | -0.6293 | 0.00004994 | 0.04286 | yes | down |
| gene21924 | *GATA2* | 0.604 | -0.7271 | 0.00005683 | 0.04286 | yes | down |
| gene3102 | *BCL2L15* | 0.514 | -0.9601 | 3.244E-07 | 0.001312 | yes | down |
| gene16619 | *RHBDD3* | 0.647 | -0.6276 | 0.00002867 | 0.03093 | yes | down |
| gene19192 | *TMEM88* | 0.649 | -0.6233 | 0.00009418 | 0.05265 | yes | down |
| gene17434 | *LOC102188339* | 1.819 | 0.8633 | 0.000001888 | 0.00509 | yes | up |
| gene23849 | *ZNF771* | 0.63 | -0.6677 | 0.0002196 | 0.09349 | yes | down |
| gene27988 | *EPPK1* | 1.505 | 0.5894 | 0.00009122 | 0.05265 | yes | up |
| gene2291 | *C3H2orf82* | 0.605 | -0.725 | 0.00005352 | 0.04286 | yes | down |
| gene14593 | *C15H11orf31* | 0.497 | -1.008 | 2.988E-08 | 0.0002417 | yes | down |
| gene12466 | *TOR4A* | 0.591 | -0.7594 | 0.0000549 | 0.04286 | yes | down |
| gene11907 | *FBXO48* | 0.614 | -0.7036 | 0.0001816 | 0.08163 | yes | down |
| gene17125 | *SFRP2* | 0.632 | -0.6621 | 0.00025 | 0.09829 | yes | down |
| gene21266 | *SIVA1* | 0.622 | -0.6848 | 0.00007192 | 0.04848 | yes | down |
| gene802 | *ATP1B3* | 0.635 | -0.6553 | 0.0001241 | 0.06087 | yes | down |
| gene13386 | *SOX18* | 0.64 | -0.6445 | 0.0001228 | 0.06087 | yes | down |
| gene7090 | *EFNA5* | 0.632 | -0.6624 | 0.0002074 | 0.09068 | yes | down |
| gene22061 | *PSMG4* | 0.612 | -0.709 | 0.000000168 | 0.0009062 | yes | down |
| gene13507 | *TCF15* | 0.592 | -0.7565 | 0.00005829 | 0.04286 | yes | down |
| gene4606 | *MALSU1* | 0.622 | -0.6847 | 0.00001961 | 0.02884 | yes | down |
| gene27673 | *LOC108634705* | 0.558 | -0.8419 | 0.000002677 | 0.006187 | yes | down |
| gene17432 | *LOC102188618* | 1.684 | 0.7517 | 0.00006243 | 0.04391 | yes | up |
| gene13023 | *CCDC3* | 0.614 | -0.7026 | 0.00003802 | 0.03845 | yes | down |
| gene1003 | *YBEY* | 0.608 | -0.7173 | 0.000005051 | 0.009081 | yes | down |
| gene17527 | *RRAD* | 1.815 | 0.8602 | 0.000000462 | 0.001495 | yes | up |
| **HRDS vs. LRDS** | |  | | | | | |
| gene25371 | *CCSAP* | 1.546 | 0.6285 | 0.000246 | 0.07386 | yes | up |
| gene27985 | *GLA* | 0.585 | -0.774 | 0.0005158 | 0.09703 | yes | down |
| gene17492 | *NDRG4* | 1.664 | 0.7346 | 0.0002817 | 0.07386 | yes | up |
| gene11888 | *SLC1A4* | 0.644 | -0.6358 | 0.0004719 | 0.09127 | yes | down |
| gene13610 | *ACSS2* | 0.602 | -0.7318 | 0.00001918 | 0.02243 | yes | down |
| gene1902 | *ITGB6* | 1.824 | 0.8674 | 0.0001605 | 0.0704 | yes | up |
| gene25434 | *ME3* | 1.671 | 0.7407 | 0.00005117 | 0.03478 | yes | up |
| gene24037 | *IFT22* | 1.525 | 0.609 | 0.0005599 | 0.09945 | yes | up |
| gene18510 | *RDH13* | 0.627 | -0.6743 | 0.0003133 | 0.07597 | yes | down |
| gene11938 | *EHD3* | 0.574 | -0.8015 | 0.0003551 | 0.07597 | yes | down |
| gene5796 | *EMP1* | 0.567 | -0.8186 | 0.0000188 | 0.02243 | yes | down |
| gene17323 | *MVD* | 0.614 | -0.7044 | 0.00005779 | 0.03526 | yes | down |
| gene6385 | *LOC106502176* | 1.781 | 0.8323 | 0.0003574 | 0.07597 | yes | up |
| gene155 | *INSIG1* | 0.598 | -0.7419 | 0.000009858 | 0.01537 | yes | down |
| gene3828 | *SLC5A3* | 0.585 | -0.774 | 0.0003284 | 0.07597 | yes | down |
| gene26110 | *LOC102185156* | 0.635 | -0.6544 | 0.00009961 | 0.0482 | yes | down |
| gene25474 | *USP35* | 0.564 | -0.8263 | 0.0002206 | 0.07052 | yes | down |
| gene2268 | *TRPM8* | 1.731 | 0.7916 | 0.0004748 | 0.09127 | yes | up |
| gene570 | *KCNMB3* | 1.606 | 0.6838 | 0.0003071 | 0.07597 | yes | up |
| gene17434 | *LOC102188339* | 2.894 | 1.533 | 1.323E-11 | 1.857E-07 | yes | up |
| gene382 | *TFRC* | 1.772 | 0.8251 | 0.0002078 | 0.07052 | yes | up |
| gene7254 | *CYFIP2* | 0.61 | -0.7133 | 0.0002695 | 0.07386 | yes | down |
| gene13657 | *RPN2* | 0.609 | -0.7149 | 0.0002104 | 0.07052 | yes | down |
| gene9788 | *PNPLA3* | 0.652 | -0.6168 | 0.0001112 | 0.05203 | yes | down |
| gene6202 | *MANEA* | 0.419 | -1.255 | 7.471E-09 | 0.00005242 | yes | down |
| gene18241 | *ATF5* | 0.618 | -0.6949 | 0.0004601 | 0.09127 | yes | down |
| gene5074 | *DDN* | 1.682 | 0.7498 | 0.0002842 | 0.07386 | yes | up |
| gene334 | *LOC108635177* | 0.574 | -0.8015 | 0.000362 | 0.07597 | yes | down |
| gene28430 | *RCAN2* | 0.557 | -0.8446 | 0.0002591 | 0.07386 | yes | down |
| gene22674 | *LOC102184282* | 1.897 | 0.9234 | 0.00007148 | 0.04012 | yes | up |
| gene5295 | *KIF13B* | 0.618 | -0.6941 | 0.0000308 | 0.03087 | yes | down |
| gene8684 | *MARS* | 0.663 | -0.5923 | 0.00000419 | 0.0098 | yes | down |
| gene23606 | *CLEC16A* | 0.657 | -0.6056 | 0.0005369 | 0.09785 | yes | down |
| gene12289 | *CERCAM* | 1.768 | 0.8222 | 0.0002211 | 0.07052 | yes | up |
| gene10205 | *SEL1L* | 0.535 | -0.902 | 9.721E-07 | 0.003 | yes | down |
| gene14137 | *LY96* | 1.582 | 0.6615 | 0.0003593 | 0.07597 | yes | up |
| gene12858 | *CYSLTR2* | 0.465 | -1.106 | 0.000001069 | 0.003 | yes | down |
| gene18543 | *LOC102172431* | 1.783 | 0.8342 | 0.0001883 | 0.07052 | yes | up |
| gene17394 | *FTH1* | 0.599 | -0.7384 | 0.00005342 | 0.03478 | yes | down |
| gene25831 | *GPT2* | 1.619 | 0.6953 | 0.0002707 | 0.07386 | yes | up |
| gene12746 | *SLC7A1* | 0.559 | -0.8391 | 0.00001261 | 0.01769 | yes | down |
| gene9219 | *LOC102188855* | 0.485 | -1.045 | 7.347E-08 | 0.0003437 | yes | down |
| gene27159 | *LOC108634447* | 1.779 | 0.8311 | 0.0003548 | 0.07597 | yes | up |
| gene3526 | *CRABP2* | 1.901 | 0.9269 | 0.00005383 | 0.03478 | yes | up |
| gene6243 | *LOC102184901* | 0.623 | -0.6825 | 0.0002145 | 0.07052 | yes | down |
| gene2648 | *DHCR24* | 0.591 | -0.7597 | 0.00004538 | 0.03478 | yes | down |
| gene22088 | *CARMIL1* | 0.66 | -0.599 | 0.00005167 | 0.03478 | yes | down |
| gene21901 | *XPC* | 1.564 | 0.6448 | 0.00007577 | 0.04089 | yes | up |
| gene10342 | *PLEKHD1* | 0.542 | -0.8835 | 0.000009337 | 0.01537 | yes | down |
| gene8833 | *ADAMTSL1* | 0.59 | -0.7623 | 0.0004368 | 0.09013 | yes | down |
| **HRDS vs. MRDS** | |  | | | | | |
| gene28865 | *LOC106503395* | 1.717 | 0.7801 | 0.0006861 | 0.06113 | yes | up |
| gene26108 | *NUDT8* | 1.647 | 0.7196 | 0.0009647 | 0.06936 | yes | up |
| gene11827 | *LOC108637084* | 1.702 | 0.767 | 0.001881 | 0.09537 | yes | up |
| gene27370 | *LOC102174372* | 0.508 | -0.9777 | 0.00002416 | 0.007947 | yes | down |
| gene21831 | *CCR5* | 0.573 | -0.8045 | 0.0008639 | 0.06691 | yes | down |
| gene7046 | *MRPL57* | 0.629 | -0.6684 | 0.001981 | 0.0974 | yes | down |
| gene12699 | *HTT* | 2.039 | 1.028 | 0.00001403 | 0.00548 | yes | up |
| gene20523 | *MRPL36* | 1.976 | 0.9828 | 0.00001181 | 0.004893 | yes | up |
| gene23329 | *MPG* | 1.595 | 0.674 | 0.0004408 | 0.04791 | yes | up |
| gene7546 | *SKP1* | 2.166 | 1.115 | 0.000004316 | 0.002809 | yes | up |
| gene9595 | *ECHDC1* | 0.665 | -0.5879 | 0.000001016 | 0.000868 | yes | down |
| gene18889 | *RAB11FIP4* | 0.591 | -0.7594 | 0.001998 | 0.09758 | yes | down |
| gene18198 | *SNRNP70* | 1.507 | 0.5919 | 0.0002885 | 0.04023 | yes | up |
| gene17525 | *PDP2* | 0.613 | -0.7053 | 0.0003575 | 0.04328 | yes | down |
| gene15277 | *PRKCDBP* | 1.574 | 0.6541 | 0.001305 | 0.08348 | yes | up |
| gene217 | *NXPE3* | 1.645 | 0.7184 | 0.001836 | 0.09502 | yes | up |
| gene14426 | *FBXL6* | 1.517 | 0.6009 | 0.0001808 | 0.0309 | yes | up |
| gene18470 | *ZNF581* | 1.643 | 0.7164 | 0.001019 | 0.07069 | yes | up |
| gene15592 | *LRRC26* | 1.708 | 0.772 | 0.0009632 | 0.06936 | yes | up |
| gene12480 | *LAYN* | 2.323 | 1.216 | 6.141E-07 | 0.0006793 | yes | up |
| gene6112 | *DNAL4* | 1.63 | 0.7051 | 0.0001515 | 0.0269 | yes | up |
| gene13640 | *MYL9* | 1.766 | 0.8207 | 0.00001057 | 0.004817 | yes | up |
| gene2406 | *CITED4* | 1.822 | 0.8655 | 0.00008743 | 0.01811 | yes | up |
| gene6264 | *LOC108636123* | 0.619 | -0.6912 | 0.001414 | 0.08587 | yes | down |
| gene24698 | *LOC102174361* | 1.66 | 0.7309 | 0.0008143 | 0.06539 | yes | up |
| gene9578 | *IBTK* | 0.649 | -0.6233 | 0.00008285 | 0.01742 | yes | down |
| gene10682 | *NEMF* | 0.659 | -0.6027 | 0.00007804 | 0.01667 | yes | down |
| gene21594 | *PDE12* | 0.648 | -0.6265 | 0.0002418 | 0.03713 | yes | down |
| gene8535 | *HOMER3* | 1.611 | 0.6883 | 0.0005529 | 0.05443 | yes | up |
| gene901 | *SH3BGR* | 1.656 | 0.7275 | 0.001137 | 0.07652 | yes | up |
| gene1484 | *STK11IP* | 0.608 | -0.7181 | 0.0004417 | 0.04791 | yes | down |
| gene13808 | *TUSC1* | 0.657 | -0.6061 | 0.002068 | 0.09925 | yes | down |
| gene8746 | *ARFGEF2* | 1.993 | 0.995 | 0.000002618 | 0.001883 | yes | up |
| gene7553 | *LOC102178707* | 0.651 | -0.6191 | 8.066E-09 | 0.00002205 | yes | down |
| gene26025 | *HSPA4* | 0.569 | -0.8128 | 0.0007568 | 0.06376 | yes | down |
| gene27692 | *CLDN5* | 1.774 | 0.8269 | 0.0006291 | 0.05736 | yes | up |
| gene18510 | *RDH13* | 0.6 | -0.738 | 0.0001923 | 0.03244 | yes | down |
| gene22604 | *LOC102190745* | 0.616 | -0.6979 | 0.001079 | 0.07413 | yes | down |
| gene18826 | *LOC102169855* | 1.505 | 0.5895 | 0.0004879 | 0.05091 | yes | up |
| gene14827 | *HSD17B12* | 0.618 | -0.6933 | 0.000001717 | 0.001303 | yes | down |
| gene18000 | *ZNF575* | 2.365 | 1.242 | 1.604E-09 | 0.000007307 | yes | up |
| gene2029 | *AGPS* | 0.643 | -0.6372 | 0.001508 | 0.08842 | yes | down |
| gene1096 | *CROCC* | 1.511 | 0.5956 | 0.001689 | 0.09125 | yes | up |
| gene19444 | *HOXB2* | 2.029 | 1.021 | 0.00003235 | 0.009409 | yes | up |
| gene18139 | *LOC108638116* | 1.854 | 0.8907 | 0.00003552 | 0.009908 | yes | up |
| gene21924 | *GATA2* | 1.945 | 0.9598 | 0.00002047 | 0.007174 | yes | up |
| gene1493 | *LOC108635183* | 1.604 | 0.6814 | 0.00007524 | 0.01659 | yes | up |
| gene28438 | *LOC102179869* | 1.586 | 0.6654 | 0.0008633 | 0.06691 | yes | up |
| gene15900 | *SPEG* | 1.715 | 0.7782 | 0.0007921 | 0.06483 | yes | up |
| gene17323 | *MVD* | 0.593 | -0.7547 | 0.000006694 | 0.003519 | yes | down |
| gene10189 | *PTPN21* | 1.552 | 0.6339 | 0.0000175 | 0.006295 | yes | up |
| gene17416 | *IRX3* | 1.616 | 0.6922 | 0.001712 | 0.09164 | yes | up |
| gene2045 | *SESTD1* | 1.58 | 0.6597 | 0.0008229 | 0.06539 | yes | up |
| gene155 | *SLC5A3* | 0.62 | -0.6885 | 0.0002173 | 0.03494 | yes | down |
| gene13635 | *SCAND1* | 1.504 | 0.5892 | 0.001405 | 0.0858 | yes | up |
| gene1483 | *SLC4A3* | 1.899 | 0.925 | 0.0001414 | 0.02577 | yes | up |
| gene3828 | *INSIG1* | 0.572 | -0.8052 | 0.0002758 | 0.03929 | yes | down |
| gene8838 | *LOC102185156* | 1.937 | 0.9538 | 0.000004988 | 0.002961 | yes | up |
| gene26110 | *CCDC85B* | 0.631 | -0.6635 | 0.0004776 | 0.05022 | yes | down |
| gene25990 | *BNC2* | 1.563 | 0.6445 | 0.0009456 | 0.06875 | yes | up |
| gene18172 | *DBP* | 1.991 | 0.9937 | 8.153E-10 | 0.000007307 | yes | up |
| gene16528 | *LOC102181889* | 1.782 | 0.8339 | 0.00001116 | 0.004893 | yes | up |
| gene17152 | *KIAA0319* | 1.502 | 0.5873 | 0.001527 | 0.08842 | yes | up |
| gene16619 | *RHBDD3* | 1.783 | 0.8344 | 3.057E-07 | 0.0003798 | yes | up |
| gene6359 | *ZGRF1* | 1.852 | 0.8894 | 0.0002088 | 0.03438 | yes | up |
| gene23816 | *YPEL3* | 1.593 | 0.6721 | 0.00002442 | 0.007947 | yes | up |
| gene20383 | *C9* | 0.653 | -0.6138 | 0.001778 | 0.0931 | yes | down |
| gene26337 | *MAGED1* | 0.638 | -0.6473 | 0.0009352 | 0.06875 | yes | down |
| gene6588 | *KLHL5* | 0.63 | -0.6664 | 0.00006892 | 0.01597 | yes | down |
| gene2879 | *PTMS* | 0.468 | -1.095 | 0.000008703 | 0.004248 | yes | down |
| gene5974 | *LOC108633177* | 1.601 | 0.6791 | 0.0001572 | 0.02754 | yes | up |
| gene24795 | *LOC108635484* | 1.886 | 0.9157 | 0.00002951 | 0.009168 | yes | up |
| gene28800 | *ZNF703* | 0.545 | -0.8762 | 0.0003259 | 0.04207 | yes | down |
| gene6202 | *PNPLA3* | 0.409 | -1.29 | 1.121E-08 | 0.00002553 | yes | down |
| gene24504 | *LOC102175922* | 1.684 | 0.7519 | 0.002131 | 0.09925 | yes | up |
| gene23849 | *ZNF771* | 1.609 | 0.6865 | 0.000752 | 0.06376 | yes | up |
| gene15454 | *SC5D* | 0.617 | -0.6956 | 0.00001176 | 0.004893 | yes | down |
| gene8254 | *GADD45GIP1* | 1.547 | 0.6291 | 0.00001651 | 0.006099 | yes | up |
| gene27988 | *EPPK1* | 1.777 | 0.8291 | 0.0005263 | 0.05409 | yes | up |
| gene15618 | *ATM* | 0.613 | -0.706 | 0.00004144 | 0.01111 | yes | down |
| gene7019 | *LOC108636239* | 1.603 | 0.6806 | 0.0004199 | 0.04743 | yes | up |
| gene17822 | *PPP1R14A* | 1.964 | 0.9738 | 0.00003155 | 0.009409 | yes | up |
| gene28879 | *LOC108635546* | 0.566 | -0.8202 | 0.0006158 | 0.05687 | yes | down |
| gene22121 | *LOC102182181* | 1.863 | 0.8977 | 0.0002317 | 0.03674 | yes | up |
| gene6298 | *LOC108636198* | 0.451 | -1.148 | 0.000003484 | 0.002381 | yes | down |
| gene6896 | *SCD5* | 1.683 | 0.7513 | 0.0004165 | 0.04743 | yes | up |
| gene22474 | *RNF39* | 1.939 | 0.9554 | 0.00002442 | 0.007947 | yes | up |
| gene25827 | *FADS2* | 1.786 | 0.8369 | 0.0007169 | 0.06281 | yes | up |
| gene22674 | *ARHGAP19* | 1.731 | 0.7913 | 0.00137 | 0.08549 | yes | up |
| gene24550 | *RCAN2* | 1.615 | 0.6914 | 0.001716 | 0.09164 | yes | up |
| gene27357 | *LOC106503400* | 0.482 | -1.052 | 0.00001459 | 0.005541 | yes | down |
| gene27270 | *LOC108634555* | 1.516 | 0.6004 | 0.00004849 | 0.01251 | yes | up |
| gene14593 | *C15H11orf31* | 2.263 | 1.178 | 2.306E-09 | 0.00000788 | yes | up |
| gene14825 | *C15H11orf96* | 1.754 | 0.8104 | 0.0007903 | 0.06483 | yes | up |
| gene18050 | *APOE* | 1.817 | 0.8613 | 3.052E-07 | 0.0003798 | yes | up |
| gene18113 | *DACT3* | 1.993 | 0.9947 | 0.0000122 | 0.004902 | yes | up |
| gene23580 | *LOC102170015* | 0.522 | -0.9378 | 0.00003662 | 0.01001 | yes | down |
| gene11801 | *ATOH8* | 1.688 | 0.7552 | 0.0005473 | 0.05443 | yes | up |
| gene15459 | *GRIK4* | 0.585 | -0.7745 | 0.0008117 | 0.06539 | yes | down |
| gene4736 | *LOC102177400* | 0.626 | -0.6753 | 0.001663 | 0.09093 | yes | down |
| gene21266 | *SIVA1* | 1.62 | 0.6961 | 0.0009047 | 0.06875 | yes | up |
| gene24474 | *IRX4* | 0.645 | -0.6327 | 0.0001279 | 0.02498 | yes | down |
| gene20521 | *PPRC1* | 1.689 | 0.7558 | 0.002109 | 0.09925 | yes | up |
| gene10301 | *EXOSC6* | 1.698 | 0.7642 | 0.002019 | 0.09786 | yes | up |
| gene17189 | *LOC102181962* | 1.601 | 0.6791 | 0.0003365 | 0.04259 | yes | up |
| gene13794 | *TP53RK* | 1.548 | 0.6303 | 0.0001305 | 0.025 | yes | up |
| gene19510 | *PPP1R1B* | 1.608 | 0.685 | 0.002142 | 0.09925 | yes | up |
| gene27560 | *ZNF185* | 0.506 | -0.9828 | 0.00005486 | 0.01389 | yes | down |
| gene24145 | *BRI3* | 1.537 | 0.62 | 0.0002984 | 0.0412 | yes | up |
| gene15989 | *CHML* | 0.567 | -0.819 | 0.0002707 | 0.03929 | yes | down |
| gene4589 | *TMEM243* | 1.629 | 0.7041 | 0.001363 | 0.08548 | yes | up |
| gene23606 | *CLEC16A* | 0.661 | -0.5978 | 0.0003261 | 0.04207 | yes | down |
| gene7524 | *CXCL14* | 1.662 | 0.7331 | 0.0009779 | 0.06936 | yes | up |
| gene21627 | *CLEC14A* | 1.629 | 0.7036 | 4.691E-08 | 0.0000916 | yes | up |
| gene20989 | *MUSTN1* | 1.526 | 0.6097 | 0.0007697 | 0.06376 | yes | up |
| gene10205 | *SEL1L* | 0.661 | -0.5967 | 0.0005707 | 0.05443 | yes | down |
| gene21047 | *GPR68* | 0.524 | -0.9311 | 2.805E-07 | 0.0003798 | yes | down |
| gene23900 | *TGFB1I1* | 1.506 | 0.5908 | 0.0003567 | 0.04328 | yes | up |
| gene28908 | *ND1* | 1.582 | 0.6618 | 0.001696 | 0.09125 | yes | up |
| gene12761 | *HSPH1* | 0.64 | -0.6448 | 0.0009211 | 0.06875 | yes | down |
| gene8192 | *ZNF358* | 1.522 | 0.606 | 0.001864 | 0.09508 | yes | up |
| gene16173 | *FAAP20* | 1.515 | 0.5992 | 0.000007751 | 0.003924 | yes | up |
| gene12858 | *CYSLTR2* | 0.452 | -1.145 | 6.958E-07 | 0.0006793 | yes | down |
| gene22306 | *LOC108633410* | 0.597 | -0.7451 | 0.001571 | 0.08873 | yes | down |
| gene3753 | *HSD17B7* | 0.576 | -0.7952 | 0.00003197 | 0.009409 | yes | down |
| gene22061 | *PSMG4* | 1.922 | 0.9429 | 1.475E-09 | 0.000007307 | yes | up |
| gene12746 | *LDLR* | 0.653 | -0.614 | 0.0008031 | 0.06534 | yes | down |
| gene8158 | *SLC7A1* | 0.573 | -0.8034 | 0.0002878 | 0.04023 | yes | down |
| gene13507 | *TCF15* | 1.828 | 0.8702 | 0.0003862 | 0.04512 | yes | up |
| gene5178 | *SLC2A13* | 0.648 | -0.6259 | 0.000763 | 0.06376 | yes | down |
| gene9219 | *MALSU1* | 0.604 | -0.7268 | 0.001207 | 0.0797 | yes | down |
| gene4606 | *LOC102188855* | 1.616 | 0.6926 | 0.00009246 | 0.01886 | yes | up |
| gene1144 | *CAMK2N1* | 1.659 | 0.7299 | 0.001339 | 0.08475 | yes | up |
| gene7100 | *NUDT12* | 0.608 | -0.7168 | 0.0009066 | 0.06875 | yes | down |
| gene5029 | *LOC102168424* | 1.687 | 0.7544 | 0.0009794 | 0.06936 | yes | up |
| gene2384 | *MFSD2A* | 0.553 | -0.8545 | 0.00003499 | 0.009908 | yes | down |
| gene17553 | *ZDHHC1* | 1.519 | 0.603 | 0.0009366 | 0.06875 | yes | up |
| gene11132 | *LOC108637011* | 1.63 | 0.7053 | 0.0005467 | 0.05443 | yes | up |
| gene10289 | *ENTPD5* | 0.633 | -0.6588 | 0.001775 | 0.0931 | yes | down |
| gene19321 | *SLC47A1* | 0.49 | -1.03 | 0.00002609 | 0.008293 | yes | down |
| gene25952 | *ZNHIT2* | 1.762 | 0.8175 | 0.00006546 | 0.01543 | yes | up |
| gene4572 | *CACNA2D1* | 1.552 | 0.6339 | 0.001612 | 0.0897 | yes | up |
| gene27673 | *LOC108634705* | 2.085 | 1.06 | 0.000000671 | 0.0006793 | yes | up |
| gene14091 | *NOV* | 1.7 | 0.7653 | 0.001855 | 0.09508 | yes | up |
| gene19391 | *CHAD* | 1.802 | 0.8494 | 0.0005591 | 0.05443 | yes | up |
| gene8272 | *C11H9orf142* | 1.577 | 0.6571 | 0.000006472 | 0.003519 | yes | up |
| gene12428 | *C7H19orf43* | 1.523 | 0.6071 | 0.0003262 | 0.04207 | yes | up |
| gene11471 | *SORBS2* | 1.548 | 0.6308 | 8.044E-07 | 0.000733 | yes | up |
| gene24908 | *INO80B* | 1.529 | 0.6125 | 0.0000052 | 0.002961 | yes | up |
| gene3848 | *SMARCD3* | 1.633 | 0.7074 | 0.00004704 | 0.01237 | yes | up |
| gene17123 | *TLR2* | 0.614 | -0.7045 | 0.0006295 | 0.05736 | yes | down |
| gene27829 | *LOC108634775* | 1.787 | 0.8376 | 0.0003588 | 0.04328 | yes | up |
| gene28842 | *LOC108635517* | 0.585 | -0.7729 | 0.0002417 | 0.03713 | yes | down |
| gene2791 | *USP33* | 0.624 | -0.6801 | 0.001437 | 0.08651 | yes | down |
| gene24017 | *HSPB1* | 1.699 | 0.7648 | 0.00005888 | 0.01437 | yes | up |
| gene13023 | *CCDC3* | 1.612 | 0.689 | 0.0006823 | 0.06113 | yes | up |
| gene12578 | *LOC102185549* | 0.51 | -0.9727 | 0.00007779 | 0.01667 | yes | down |
| gene2648 | *DHCR24* | 0.646 | -0.6314 | 0.001646 | 0.09037 | yes | down |
| gene25145 | *MSS51* | 1.665 | 0.7353 | 0.001973 | 0.09734 | yes | up |
| gene18474 | *ZNF524* | 1.507 | 0.592 | 0.001681 | 0.09125 | yes | up |
| gene19475 | *SOCS7* | 0.589 | -0.763 | 0.001561 | 0.08853 | yes | down |
| gene9238 | *ENHO* | 1.624 | 0.6996 | 0.001554 | 0.08853 | yes | up |
| gene8282 | *LOC102183952* | 0.563 | -0.8292 | 0.0006887 | 0.06113 | yes | down |
| gene10342 | *PLEKHD1* | 0.487 | -1.037 | 0.000001215 | 0.0009768 | yes | down |
| gene5130 | *TMEM106C* | 0.654 | -0.6125 | 0.00007452 | 0.01659 | yes | down |
| gene28758 | *LOC108635449* | 0.544 | -0.877 | 0.0003948 | 0.04534 | yes | down |
| gene3912 | *BBIP1* | 1.611 | 0.6883 | 0.001831 | 0.09502 | yes | up |
| gene24410 | *LOC102190581* | 1.59 | 0.6692 | 0.0004607 | 0.0492 | yes | up |
| gene26219 | *ATRX* | 0.56 | -0.8359 | 0.0005442 | 0.05443 | yes | down |

^1^FDR-value: FDR-value is the P value corrected by Benjamini-Hochberg correction to account for false discovery rate.

**Table S7 Gene Ontology analysis of differentially expressed genes among there treatments.**

| Number | GO ID | Term type | Description | Ratio in study | Ratio in pop | *P*-value | FDR-value | Gene name |
| --- | --- | --- | --- | --- | --- | --- | --- | --- |
| 14 | GO:0008610 | BP | Lipid biosynthetic process | 14/177 | 321/16419 | 1.027E-05 | 0.016 | *ACSS2, MVD, AGPS, SCD5, FADS2, CERCAM, HSD17B7, HSD17B12, TP53RK, SC5D, ATM, INSIG1, LOC102177400, GDF1* |
| 4 | GO:1902653 | BP | Secondary alcohol biosynthetic process | 4/177 | 24/16419 | 0.0001172 | 0.286 | *INSIG1, MVD, HSD17B7, LOC102177400* |
| 4 | GO:0006695 | BP | Cholesterol biosynthetic process | 4/177 | 24/16419 | 0.0001172 | 0.286 | *INSIG1, MVD, HSD17B7, LOC102177400* |
| 4 | GO:0016126 | BP | Sterol biosynthetic process | 4/177 | 26/16419 | 0.0001621 | 0.338 | *INSIG1, MVD, HSD17B7, LOC102177400* |
| 4 | GO:0045599 | BP | Negative regulation of fat cell differentiation | 4/177 | 29/16419 | 0.0002512 | 0.400 | *INSIG1, GATA2, TGFB1I1, CCDC85B* |
| 3 | GO:0007186 | BP | G-protein coupled receptor signaling pathway | 3/177 | 1388/16419 | 0.0003142 | 0.444 | *GPR68, CYSLTR2, CCR5* |
| 2 | GO:0004768 | MF | Stearoyl-CoA 9-desaturase activity | 2/177 | 3/16419 | 0.0003442 | 0.472 | *SCD5, FADS2* |
| 2 | GO:0016215 | MF | acyl-CoA desaturase activity | 2/177 | 3/16419 | 0.0003442 | 0.472 | *SCD5, FADS2* |
| 5 | GO:0008203 | BP | Cholesterol metabolic process | 5/177 | 59/16419 | 0.0004302 | 0.636 | *INSIG1, MVD, HSD17B7, LDLR, LOC102177400* |
| 5 | GO:1902652 | BP | Secondary alcohol metabolic process | 5/177 | 61/16419 | 0.0005024 | 0.650 | *INSIG1, MVD, HSD17B7, LDLR, LOC102177400* |
| 5 | GO:0016125 | BP | Sterol metabolic process | 5/177 | 62/16419 | 0.0005417 | 0.672 | *INSIG1, MVD, HSD17B7, LDLR, LOC102177400* |
| 5 | GO:0045598 | BP | Regulation of fat cell differentiation | 5/177 | 65/16419 | 0.0006738 | 0.69 | *INSIG1, SFRP2, GATA2, TGFB1I1, CCDC85B* |
| 2 | GO:0007187 | BP | G-protein coupled receptor signaling pathway, Coupled to cyclic nucleotide second messenger | 2/177 | 1087/16419 | 0.0011129 | 0.872 | *CYSLTR2, CCR5* |
| 2 | GO:0042159 | BP | Lipoprotein catabolic process | 2/177 | 5/16419 | 0.0011312 | 0.872 | *ATM, LDLR* |
| 2 | GO:0042535 | BP | positive regulation of tumor Necrosis factor biosynthetic process | 2/177 | 5/16419 | 0.001131188 | 0.872 | *HSPB1, TLR2* |
| 5 | GO:0006694 | BP | Steroid biosynthetic process | 5/177 | 75/16419 | 0.001291198 | 0.936 | *INSIG1, MVD, HSD17B7, LOC102177400* |
| 3 | GO:0004930 | MF | G-protein coupled receptor activity | 3/177 | 1235/16419 | 0.001325715 | 0.936 | *GPR68, CYSLTR2, CCR5* |
| 2 | GO:0009103 | BP | Lipopolysaccharide biosynthetic process | 2/177 | 6/16419 | 0.001684755 | 0.96 | *CERCAM, TP53RK* |
| 3 | GO:0042157 | BP | Lipoprotein metabolic process | 3/177 | 23/16419 | 0.001861154 | 0.978 | ATM, APOE, LDLR |

BP means biological process.

MF means molecular function.

The population was referred to Capra-hircus (https://www.ncbi.nlm.nih.gov/genome/?term=txid9925[orgn]).

| Num | Term | Pathway ID | Database | Ratio in study | Ratio in pop | *P*-value | FDR-value | Gene name |
| --- | --- | --- | --- | --- | --- | --- | --- | --- |
| 4 | Steroid biosynthesis | map00100 | KEGG PATHWAY | 4/77 | 20/8030 | 3.37043E-05 | 0.00498824 | *DHCR24,HSD17B7, SC5D, LOC102177400* |
| 4 | Biosynthesis of unsaturated fatty acids | map01040 | KEGG PATHWAY | 4/77 | 26/8030 | 9.95581E-05 | 0.007367297 | *SCD5, FADS2, HSD17B12, LOC102181962* |
| 5 | Toxoplasmosis | map05145 | KEGG PATHWAY | 5/77 | 120/8030 | 0.005752118 | 0.283771169 | *LOC102181962, LDLR, TLR2, CCR5* |
| 3 | Mineral absorption | map04978 | KEGG PATHWAY | 3/77 | 49/8030 | 0.011385224 | 0.421253277 | *FTH1, LOC102188618, ATP1B3* |
| 5 | Protein processing in endoplasmic reticulum | map04141 | KEGG PATHWAY | 5/77 | 172/8030 | 0.02435739 | 0.720978731 | *SKP1, SEL1L, RPN2, LOC102174361, HSPH1* |
| 2 | Fatty acid elongation | map00062 | KEGG PATHWAY | 2/77 | 28/8030 | 0.029207356 | 0.720448123 | *HSD17B12, LOC102181962* |
| 2 | Propanoate metabolism | map00640 | KEGG PATHWAY | 2/77 | 33/8030 | 0.039567389 | 0.836567645 | *ACSS2,ECHDC1* |
| 2 | Pyruvate metabolism | map00620 | KEGG PATHWAY | 2/77 | 39/8030 | 0.053534133 | 0.990381458 | ACSS2, ME3 |
| 6 | Endocytosis | map04144 | KEGG PATHWAY | 6/77 | 283/8030 | 0.053629914 | 0.881914148 | *TFRC, EHD3, RAB11FIP4, ARFGEF2, LDLR, CCR5* |
| 2 | Sphingolipid metabolism | map00600 | KEGG PATHWAY | 2/77 | 49/8030 | 0.079964213 | 1 | *GLA, LOC102170015* |

**Table S8 KEGG pathways analysis of differentially expressed genes among there treatment**

The population was referred to Capra-hircus (https://www.ncbi.nlm.nih.gov/genome/?term=txid9925[orgn]).

**Supplementary Figure S1 OPLS-DA score of MRDS vs. LRDS, HRDS vs. LRDS and HRDS vs. MRDS in the positive mode and negative mode.**

Note: **a**, **b**, and **c** respectively represent the OPLS-DA score of MRDS vs. LRDS, HRDS vs. LRDS and HRDS vs. MRDS in positive mode. **d**, **e**, and **f** respectively represent the OPLS-DA score of MRDS vs. LRDS, HRDS vs. LRDS and HRDS vs. MRDS in negative mode.


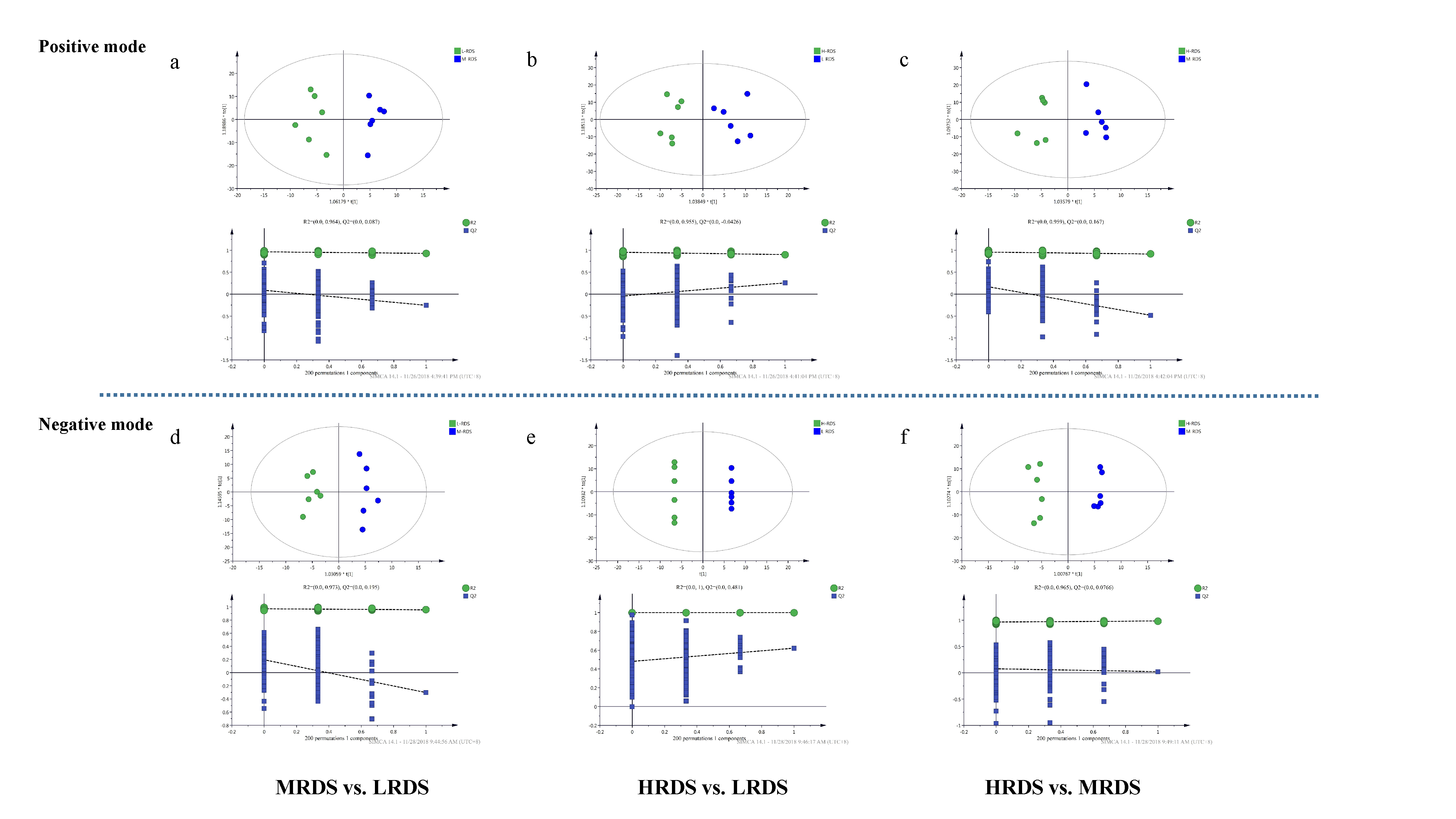

Supplement: Supplementary file 1 — Additional file 1: Table S1. Ingredients and chemical composition of diets. Table S2. The specific primers for the qRT-PCR of GAPDH and the tested mRNAs. Table S3. The specific primers used for qRT-PCR of eubacteria. Table S4. Concentration of major fatty acids in the different RDS diets. Table S5. Identified differential metabolites in MRDS vs. LRDS, HRDS vs. LRDS and HRDS vs. MRDS. Table S6. The differentially expressed genes in the compared groups of MRDS vs. LRDS, HRDS vs. LRDS and HRDS vs. MRDS. Table S7. Gene Ontology analysis of differentially expressed genes among the treatments. Table S8. KEGG pathway analysis of differentially expressed genes among the treatments. Figure S1. OPLS-DA score of MRDS vs. LRDS, HRDS vs. LRDS and HRDS vs. MRDS in positive mode and negative mode. Note: a, b, and c represent the OPLS-DA scores of MRDS vs. LRDS, HRDS vs. LRDS and HRDS vs. MRDS in positive mode, respectively. d, e, and f represent the OPLS-DA scores of MRDS vs. LRDS, HRDS vs. LRDS and HRDS vs. MRDS in negative mode, respectively. [file 40104_2020_436_MOESM1_ESM.doc]
